# A numerical algorithm for modeling cellular rearrangements in tissue morphogenesis

**DOI:** 10.1038/s42003-022-03174-6

**Published:** 2022-03-18

**Authors:** Rhudaina Z. Mohammad, Hideki Murakawa, Karel Svadlenka, Hideru Togashi

**Affiliations:** 1 Department of Mathematics, Graduate School of Science, Kyoto University, Kyoto, Japan; 2 Institute of Mathematics, College of Science, University of the Philippines Diliman, Quezon City, Philippines; 3 Applied Mathematics and Informatics Course, Faculty of Advanced Science and Technology, Ryukoku University, Otsu, Japan; 4 Mathematical Institute, Czech Academy of Sciences, Prague, Czech Republic; 5 JST PRESTO (Precursory Research for Embryonic Science and Technology), Kobe, Japan; 6 Department of Biochemistry and Molecular Biology, Kobe University Graduate School of Medicine, Kobe, Japan

**Keywords:** Cell adhesion, Pattern formation, Computational models

## Abstract

Among morphological phenomena, cellular patterns in developing sensory epithelia have gained attention in recent years. Although physical models for cellular rearrangements are well-established thanks to a large bulk of experimental work, their computational implementation lacks solid mathematical background and involves experimentally unreachable parameters. Here we introduce a level set-based computational framework as a tool to rigorously investigate evolving cellular patterns, and study its mathematical and computational properties. We illustrate that a compelling feature of the method is its ability to correctly handle complex topology changes, including frequent cell intercalations. Combining this accurate numerical scheme with an established mathematical model, we show that the proposed framework features minimum possible number of parameters and is capable of reproducing a wide range of tissue morphological phenomena, such as cell sorting, engulfment or internalization. In particular, thanks to precise mathematical treatment of cellular intercalations, this method succeeds in simulating experimentally observed development of cellular mosaic patterns in sensory epithelia.

## Abstract (web summary)

A numerical algorithm handles complex cell topology changes and reproduces tissue morphological phenomena without relying on nonphysical parameters.

## Introduction

Understanding the mechanisms of tissue morphogenesis—how interacting processes generate the shape and structure of an organism—is at the forefront of researches in developmental biology. Mathematical modeling is a powerful tool to investigate how local cell-cell interactions affect tissue-level morphology. In general, a successful model requires two main ingredients: a mathematical model represented by a set of governing equations based on physical principles, and a computational method to solve them. Building each of these two components is a different process—a simple physical model may not necessarily be easy to compute. In this paper, we focus on the computational aspect of modeling the morphology of epithelial tissues, where the gap between a model and its numerical realization is prominent. Indeed, the physical model usually measures adhesion or tension forces along cellular interfaces and thus is straightforward, while its computation presents challenging tasks due to topology changes in cellular rearrangement—a highly nontrivial problem from the viewpoint of mathematics.

Formation of cellular patterns in tissues often involves frequent neighbor swaps (topology changes). Cell intercalation, a typical process of this type, requires a distinctive combination of mechanisms, including adhesive changes that allow cells to rearrange, or cytoskeletal events through which cells exert the forces needed for cell neighbor exchange. Brodland^1^ in his Differential Interfacial Tension Hypothesis (DITH) proposed that relative intensities of interfacial tensions, which are defined as a combination of cell-cell adhesion, contraction of cell membranes and other forces, lead to self-driven rearrangement of embryonic cells. To analyze the effect of such factors in tissue self-organization, a number of mathematical models have been proposed with the assumption that tissue evolves via a succession of quasi-equilibrium states, that is, cell shapes are described by their instantaneous state of lowest energy^2^. In this article, we follow this line and focus on a class of models which neglect inertial effects and treat the evolution of a cellular aggregate from the viewpoint of free energy minimization principle; see Methods for more details.

Existing computational approaches realizing the above-mentioned free energy minimization include, among others, vertex dynamics model, cellular Potts model, front-tracking and finite element methods (see Supplementary Note 1 for a brief overview of their differences). In this paper, we present another class of numerical methods based on the implicit level set representation of the shape of cell-cell junctions. Here an evolving junction is a contour of a time-dependent function on a fixed spatial grid. Recent mathematical results on the evolution of level sets of interfacial networks via energy gradient descent^3^ allow for a precise computation of this evolution including topology changes. We draw attention to this computational method for its advantages and to show that in certain problems it may perform better than well-established schemes. Namely, we claim that besides its ability to accurately handle topology changes, the level set approach eliminates nonphysical parameters.

Although each specific morphogenetic phenomenon involves a large number of biological and physical factors, it is a well-accepted understanding in the modeling community that it is not reasonable to construct models having a large number of factors as model parameters. The reason is simply that correlation analysis becomes prohibitively complicated with increasing number of parameters, especially in living systems: if a model has a sufficiently large number of parameters, their suitable tuning can produce essentially arbitrary results and the analysis becomes pointless. This leads researchers to pin down biologically important components in a given phenomenon, and build a model with only those components as parameters. However, the aforementioned models incorporate a number of parameters which cannot be omitted and have only vague physical interpretations. For example, to deal with topological changes due to cellular intercalations one performs junctional rearrangements in the vertex dynamics model through simple operations, such as T1, T2, T3 transitions^4^; and in the finite element-based method through a boundary “flip” algorithm^5^, which all require additional parameters.

A typical example of a phenomenon where the above concerns become relevant is formation of cellular patterns in morphogenesis of sensory epithelia. These epithelia show regular mosaic or checkerboard patterns that crystallize through continuous intercalations of sensory and supporting cells (SCs). In the work of Katsunuma et al.^6^, it was hypothesized that varying adhesion strengths between participating cells is the decisive factor determining the mosaic pattern.

However, it was found that frequent occurrence of cell intercalations precludes the application of vertex dynamics method to support the hypothesis through simulations. Our proposed level set scheme addresses all the main causes of failure of vertex dynamics in this case, namely, inaccurate handling of topology changes, presence of nonphysical parameters, and inability of expressing complex curved shapes of cell junctions with largely different cell sizes, leading to successful reproduction of experimentally observed cellular patterns. Our results give a strong indication of the correctness of the hypothesis that differential interfacial tension plays the main role in formation of epithelial tissues.

## Results

### Mathematical model and level set-based approach

We present an outline of the mathematical model and numerical algorithm (see Methods for a systematic explanation). In choosing a model, we adhere to the context of DITH^1^. Cells in an aggregate are expressed as closed regions ${{{{{{{{\mathcal{C}}}}}}}}}_{1},\ldots ,{{{{{{{{\mathcal{C}}}}}}}}}_{N}$, and cell-cell junctions are defined by ${\gamma }_{ij}:={{{{{{{{\mathcal{C}}}}}}}}}_{i}\cap {{{{{{{{\mathcal{C}}}}}}}}}_{j}$. In cases where there is an extracellular space, we denote this medium by the same symbol ${{{{{{{{\mathcal{C}}}}}}}}}_{k}$ and its cell-medium interface by *γ*_*ik*_. Surface tension between *i*th and *j*th cell, that is equivalent to interfacial free energy density, is denoted by *σ*_*ij*_ (see Supplementary Note 2 for a discussion on its physical meaning). We consider cellular rearrangement as the *L*^2^-gradient flow of the free energy

1 $$E({{{{{{{{\mathcal{C}}}}}}}}}_{1},\ldots ,{{{{{{{{\mathcal{C}}}}}}}}}_{N})=\mathop{\sum}\limits_{i\ne j}{\sigma }_{ij}\,{{\mbox{Area}}}\,({\gamma }_{ij})$$

constrained by each cell’s prescribed volume ${V}_{\ell }^{0}$ (*ℓ* = 1, 2, …, *N*). We assume that each cell exactly preserves its volume and that apoptosis does not occur but this simplification can be made more realistic by allowing varying volumes of cells, see Supplementary Note 2. Thus, our mathematical model is standard and conceptually simple but its numerical implementation is not obvious.

**Fig. 1:**
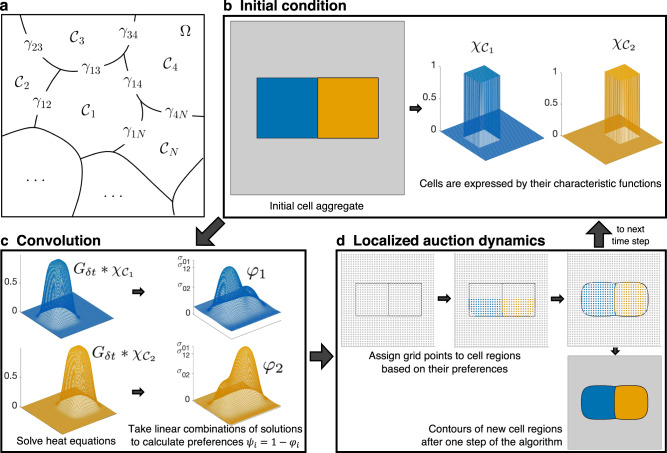
Basic notation and outline of the algorithm. **a** A cellular aggregate is represented as a bounded domain Ω partitioned into *N* cells ${{{{{{{{\mathcal{C}}}}}}}}}_{1},\ldots ,{{{{{{{{\mathcal{C}}}}}}}}}_{N}$. The boundary between cells ${{{{{{{{\mathcal{C}}}}}}}}}_{i}$ and ${{{{{{{{\mathcal{C}}}}}}}}}_{j}$ denotes the cell-cell junction *γ*_*ij*_. **b**–**d** Three main steps of the algorithm generating an approximation of the cellular rearrangement at the next time step *δt*: Initial condition (**b**). Given an initial cell aggregate, it is represented by characteristic functions ${\chi }_{{{{{{{{{\mathcal{C}}}}}}}}}_{i}}$, *i* = 1, …, *N*, of respective cell regions on a discrete grid (functions corresponding to the gray region are not shown). Convolution (**c**). Characteristic functions are diffused via solving heat equation, that is, convolutions with Gaussian kernel ${G}_{\delta t}* {\chi }_{{{{{{{{{\mathcal{C}}}}}}}}}_{i}}$ are computed for every *i*, followed by linear combination of diffused functions with weights corresponding to interfacial tensions *σ*_*ij*_, i.e., ${\varphi }_{i}=\mathop{\sum }\nolimits_{j = 1}^{N}{\sigma }_{ij}({G}_{\delta t}* {\chi }_{{{{{{{{{\mathcal{C}}}}}}}}}_{j}})$. The values *ψ*_*i*_(*x*) = 1 − *φ*_*i*_(*x*) then determine the preference of each (grid) node *x* for belonging to cell region ${{{{{{{{\mathcal{C}}}}}}}}}_{i}$. Localized auction dynamics (**d**). Grid points are assigned to cell regions in several sweeps of auction-like process where grid points bid to neighboring cells based on the preferences *ψ*_*i*_. Level sets of the final bid *b*(*x*) determine the cell configuration at the next time step *δt*, which optimizes preferences while preserving cell volumes (see also Supplementary Movie 5).

We propose a level set-based scheme for numerical realization of this model, based on the Esedo${\bar{\rm g}}$lu-Otto algorithm^3^. The main idea is to solve heat equations and to extract level sets of their solutions, which can be easily and efficiently implemented. Moreover, the convergence of this algorithm is supported by Laux and Otto^7^ from mathematical point of view even for the problem containing multiple junctions. In order to apply Esedo${\bar{\rm g}}$lu-Otto algorithm to simulations of evolving cell aggregates, two issues need to be addressed: volumes of cells have to be preserved or controlled, and cells have to be prevented from splitting. We have implemented volume control by combining the Esedo${\bar{\rm g}}$lu-Otto algorithm with auction dynamics^8^, which in essence finds suitable contour heights by simulating an auction (see Fig. 1 and the intuitive explanation of the auction algorithm in Methods). The second issue relates to the absence of any device in the original algorithm that would keep cells as connected sets. It may then happen that a cell splits into two parts and one part suddenly appears in a distant part of the aggregate, see Supplementary Note 3 for more. To avoid such behavior, we implement a localization on the bidding process in auction dynamics. Figure 1 depicts the main steps of the resulting algorithm.

In Methods, we provide further details of the algorithm, summarize its known mathematical properties such as stability and convergence, and give a detailed account of parameters used. The important conclusion is that outputs of the proposed algorithm depend only on the physical parameters *σ*_*ij*_, apart from the unavoidable space-time discretization steps *δx*, *δt*. Since theoretical results on convergence and stability for the full volume-controlled problem including localization are not available, we also present a computational analysis of the proposed method showing that it has the desired properties, in particular convergence even across topology changes. Moreover, we perform a series of numerical tests to contrast the properties of our level set-based algorithm with those of the vertex dynamics algorithm, a commonly-used method for cell intercalations in developmental biology, showing that the level set-based method yields better results at the price of higher computational cost. We defer the details on the setup and results of these experiments to Supplementary Notes 4, 5 and 6.

In Supplementary Note 7, we report that our level set-based method generates expected results for standard morphogenetic benchmarks, namely cell sorting, mixing and formation of checkerboard or football patterns. Finally, in Supplementary Note 8, performing a simulation of an encapsulation phenomenon in embryo morphogenesis, we show that our method can handle simulations in higher dimensions without major technical or theoretical complications.

### Simulation of tissue engulfment

**Fig. 2:**
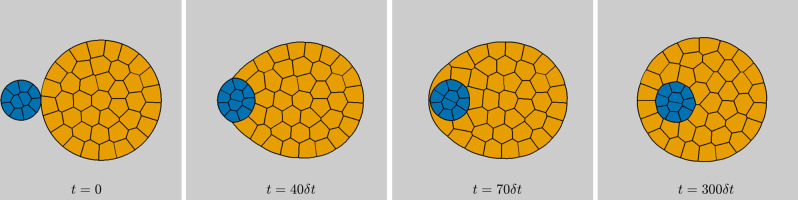
Total engulfment via level set-based model. An initial aggregate of 10 blue and 40 orange cells surrounded by a gray medium and snapshots of its evolution generated by our level set-based method, resulting in total engulfment of the blue cell mass by the orange cell mass. Here the interfacial tensions are *σ*_BB_ = *σ*_OO_ = 1.0, *σ*_BG_ = 7.5, *σ*_OG_ = 3.5, and *σ*_BO_ linearly changing from 7.5 at time *t* = 0 to 2.5 at time *t* = 100*δt* and then kept constant.

With the purpose of further validating the proposed method from the biological viewpoint, we present an application to a morphogenetic phenomenon in a medium, where one cell mass, say orange, totally engulfs another cell mass, say blue. Following the viewpoint of DITH, Brodland and Chen^9^ proposed that for the blue cell mass to be enveloped by the orange cell mass, a sufficient condition is *σ*_BO_ < *σ*_BG_ − *σ*_OG_ (here *σ*_BO_ means the interfacial tension between blue and orange cells; analogously for *σ*_BG_, *σ*_OG_, where G means the gray medium). Moreover, for such engulfment to continue, it is necessary to have *σ*_BO_ < *σ*_BG_^1^. With this in mind, consider an initial configuration of 10 blue cells, 40 orange cells, and a gray medium on a computational domain Ω = [0, 1] × [0, 1] discretized uniformly into *M* = 500 × 500 points with periodic boundary conditions. We take interfacial tensions *σ*_BB_ = *σ*_OO_ = 1.0, *σ*_BG_ = 7.5, *σ*_OG_ = 3.5, *σ*_BO_ = 7.5, then linearly change *σ*_BO_ from 7.5 to 2.5 over 100 time steps, keeping *σ*_BO_ = 2.5 for the remaining time of the simulation, cf., Brodland^1^. We then generate the corresponding aggregate evolution using our level set-based scheme with time step *δt* = 0.001. Observe that the final configuration results in total engulfment of the blue cell mass by the orange cell mass (Fig. 2 and Supplementary Movie 1^10^). This is not easily achieved by finite element-based simulations, cf., Fig. 8 of Brodland^1^, which yield unnaturally distorted shapes of cells, particularly, in the region where the blue cell mass is engulfed by orange cells. The artifacts in the FEM-based approach emerge from explicit treatment of cellular intercalations using an ad hoc boundary “flip” algorithm. This demonstrates the superiority of our method over the finite element-based scheme in handling such topology changes.

### Simulation of cellular rearrangement in olfactory epithelia

In the following two sections, we introduce simulation results showing the potential of the level set method in computationally reproducing observed cellular patterns in developing sensory systems, in particular, olfactory and auditory epithelia. Such morphologies involve curved cell junctions, largely different sizes of participating cells and frequent topology changes. The level set approach is successful in dealing with this complexity not only because it allows for a wide range of geometrical patterns but also because it implicitly satisfies the precise contact angles at tricellular junctions, which is indispensable for theoretically correct realization of cellular intercalation processes.

Vertebrates possess highly developed sense organs, responsible for detecting information about different environments and converting extracellular stimuli into electrical signals which are mediated by specialized sensory epithelia^11^. Mosaic cellular patterns have been observed in various sensory epithelia, such as football pattern in the olfactory epithelium (OE) and checkerboard pattern in the auditory epithelium. These regular mosaic patterns are evolutionary conserved among a wide range of species and thought to be important in sensory functions.

**Fig. 3:**
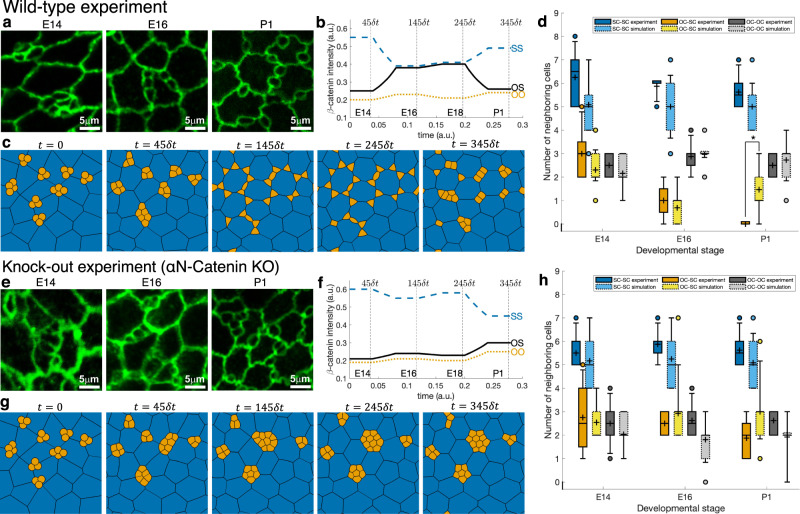
Actual images and simulation results of a developing olfactory epithelium. **a** Immunostaining for junctional marker (ZO-1) on the apical surface of the mouse OE from embryonic days (E14, E16) to postnatal day (P1) (reproduced from Journal of Cell Biology, ⓒ2016 Katsunuma et al.^6^). Scale bar 5 *μ*m (all figures have the same scale). **b** Plot of relative intensity of *β*-catenin accumulations at OO (orange dotted line), OS (solid black line), and SS (blue dashed line) junctions during development, obtained by interpolation of *β*-catenin intensity mean values at time instants E14, E16, E18, and P1 reported in Fig. S2 of Katsunuma et al.^6^. Moreover, the initial (E14) and final (P1) values were kept constant until the cellular pattern equilibrated (Supplementary Data 1). **c** A simulation of cellular rearrangement of an olfactory epithelium from initial aggregate of 26 OCs (orange) and 24 SCs (blue) to embryonic stages E14, E16, E18 until postnatal P1 stage obtained by the level set-based model. **d** Cell neighbor counts obtained from three representative actual images and one simulation image for *t* = 45*δt*, 145*δt* and 345*δt* shown in (**c**). The boxes in boxplots show first and third quartiles, whiskers show 9th and 91th percentiles and + sign shows the mean. Raw data are provided in Supplementary Data 2. Significant difference (independent *t*-test with *p* = 0.0002, marked by asterisk) was found in OC-OC neighbors at P1 stage (*n* = 8 (experiment); *n* = 24 or *n* = 26 (simulation)). **e**–**h** Corresponding results for *α*N-catenin KO mouse model based on experimentally measured *β*-catenin intensities.

The OE, which is located inside the nasal cavity, is a specialized sensory epithelium that is involved in odor perception. When the luminal surface of the OE is observed from the apical side, ciliated olfactory cells (OCs) and several types of SCs are arranged in a unique mosaic pattern (Fig. 3a). In a developing OE, this pattern formation is accompanied by cellular rearrangements from embryonic day 14 (E14) to postnatal day 1 (P1) (Fig. 3a), which is thought to be driven by the different adhesion between OCs and SCs^6^. To estimate cell-cell adhesion strength, we use the distributional patterns of *β*-catenin intensities since in this case, cadherin-dependent affinity is the major component of cell-cell adhesion. During the rearrangement, adhesion strengths between OCs and SCs (OS), SCs and SCs (SS), and OCs and OCs (OO) continuously change (Fig. 3b).

Using distributional patterns of experimentally measured *β*-catenin intensities, cf. Katsunuma et al.^6^, we apply our level set-based approach to simulate cellular rearrangements in the OE from E14 to P1 stage (Fig. 3c). We consider an initial aggregate of 26 OCs (orange) and 24 SCs (blue) on a square domain Ω = [0, 1] × [0, 1] with periodic boundary conditions. As observed in biological experiments, OCs are approximately 10 times smaller in size than SCs and tend to cluster around tricellular SC-SC junctions at E14 (see Fig. 3a). We discretize the domain uniformly into *M* = 500 × 500 points, take time step *δt* = 0.0008, and set interfacial tension *σ*_*ij*_ as the reciprocal of cell-cell adhesion strength *α*_*ij*_, measured in terms of *β*-catenin intensity.

**Fig. 4:**
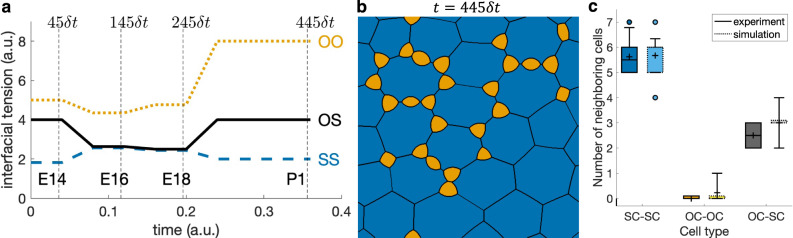
Simulation of developing olfactory epithelium until postnatal stage. **a** Evolution of interfacial tensions at OO (orange dotted line), OS (solid black line), and SS (blue dashed line) junctions based on measurements up to embryonic stage E18 but is selected hypothetically for postnatal stage P1 (Supplementary Data 3). **b** Pattern obtained at the final time 445*δt*, showing round separate olfactory cells (orange) and supporting cells (blue) arranged in a pattern similar to experimental image in Fig. 3a (P1). Patterns obtained at earlier times until 245*δt* are identical to those in Fig. 3c. **c** Boxplot of neighboring cell counts corresponding to this hypothetical simulation. Counts for the simulation image (**b**) are shown on the right in light colors. The meaning of the boxes and markers is the same as in Fig. 3d.

Figure 3c and Supplementary Movie 2^10^ show the simulation of cellular rearrangement of a developing OE. Comparing this to biological experiments, we see that the level set-based model was able to capture overall cellular rearrangements in the embryonic stage. In particular, at E14 stage, OCs cluster at the tricellular SC-SC junctions; then from E16 to E18, OCs separate and move along SC-SC junctions. The only noticeable difference is that OCs at tricellular junctions are not as round as in the experimental results. Lastly, postnatal simulation indicates that *β*-catenin and, thus, cadherin-dependent cell-cell adhesion, is not the only contributing factor for cellular rearrangement in OE. Indeed, the discrepancy observed in postnatal stages is caused by an insufficiency in the model parameters rather than by the level set-based numerical method or the model itself, since if the parameters of the model are chosen in a suitable hypothetical way, cellular patterns matching those observed experimentally are obtained by the level set approach. To generate cellular patterns similar to those observed in OE at P1 stage, we took the same initial configuration and the same discretization parameters as above. The interfacial tensions *σ*_SS_, *σ*_OS_, and *σ*_OO_ were evolved as shown in Fig. 4a; namely, until stage E18 interfacial tensions identical to those obtained from measurements as presented in Fig. 3b are adopted, while at further stages *σ*_OS_ and *σ*_OO_ were increased so that eventually *σ*_OS_ is twice larger than *σ*_SS_ and *σ*_OO_ is twice larger than *σ*_OS_. This intends to imitate the hypothesized prominent activity of cytoskeleton within OCs. The resulting evolution of the aggregate is shown in Fig. 4b and Supplementary Movie 3^10^, exhibiting a clear agreement with the actual pattern depicted in Fig. 3a (P1), which is further quantified in Fig. 4c by analyzing the numbers of neighboring cell types. In particular, OCs are separated one from another and are located either at tricellular junctions or in the middle of boundary between SCs.

To assess how well our numerical simulation captures experimentally observed results, we also consider cellular patterns observed in developing OE derived from *α*N-catenin knock-out (KO) mice. Here adhesiveness at OC-SC junctions is substantially decreased, which severely affects the resulting cellular pattern (Fig. 3e). Using distributional patterns of *β*-catenin intensities measured from *α*N-catenin KO mice, we applied our level set-based approach to simulate cellular rearrangements in the OE from E14 to P1 stage (see Fig. 3g and Supplementary Movie 2^10^). We then analyze both wild-type (WT) and KO cellular patterns by counting neighboring OCs and SCs at randomly selected cells in three actual images, and at each computational cell of the simulations shown in Fig. 3c, g. To be precise, for each olfactory cell, number of neighboring supporting cells (OC-SC) and olfactory cells (OC-OC), and for each supporting cell, number of neighboring supporting cells (SC-SC) is counted. We confirmed that the counts do not essentially depend on the initial condition by running four more simulations, starting from randomly generated initial configurations of 26 OCs and 24 SCs. Figure 3d, h reveals that the mean cell morphology for both WT and KO obtained in silico matches well with experimental counterparts in the embryonic stage, but a major discrepancy is observed in the postnatal stage. We conclude that the level-set based numerical method is able to simulate complex cell behaviors in evolving OE across all stages until P1, while the physical model based on *β*-catenin mediated adhesion captures well the phenomenon only in the embryonic stages.

### Simulation of checkerboard pattern formation in auditory epithelia

**Fig. 5:**
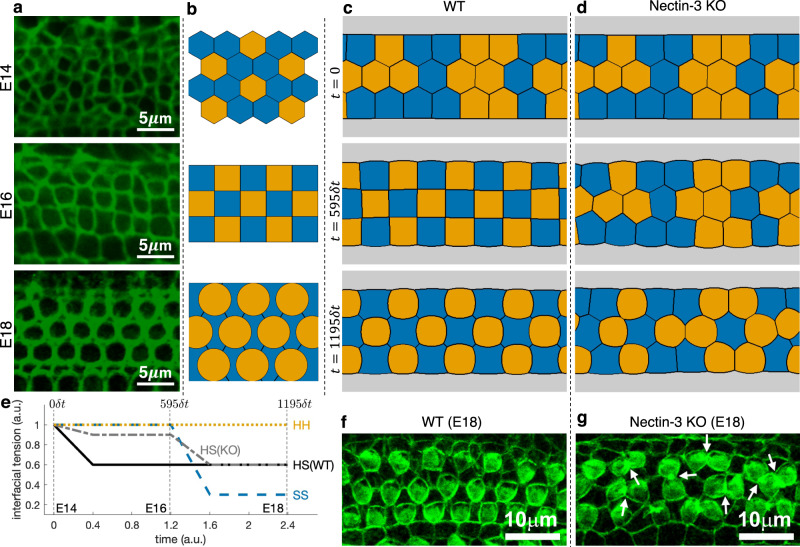
Actual images and simulation results of a developing auditory epithelium. **a** Localization of junctional marker (ZO-1) at the apical surface of the auditory epithelium at embryonic days E14, E16, and E18 (reproduced from Front. Cell Dev. Biol., ^ⓒ^2016 Togashi^11^). **b** Schema of the distribution of hair cells (HC, orange) and supporting cells (SC, blue) observed in experiments (adapted from Front. Cell Dev. Biol., ^ⓒ^2016 Togashi^11^). **c**, **d** Simulations of cellular rearrangements of WT (**c**) and Nectin-3 KO (**d**) embryonic auditory epithelium from initial aggregate of 12 HCs (orange), 12 SCs (blue), and one pillar cell (gray), generated employing level set-based model. **e** Plot of evolution of interfacial tensions at SS (blue dashed line), HS (WT: black solid line, KO: gray dash-dotted line), and HH (orange dotted line) junctions used for the simulations in (**c**, **d**) (Supplementary Data 4). **f**, **g** Actual images of auditory epithelium of WT mouse and Nectin-3 KO mouse at E18 stage, with arrows indicating aberrantly attached HCs (reproduced from Science, ^ⓒ^2011 Togashi et al.^12^).

Another example of unique cellular pattern formation in tissues is the auditory epithelium, which is responsible for hearing. It is composed of mechanosensory hair cells (HCs), equipped with stereocilia that sense sound, and SCs that help the functions of HCs. These cells rearrange to form a checkerboard pattern from embryonic day 14 (E14) to 16 (E16) (Fig. 5a, b). At E18, HCs are arranged in ordered rows, and each HC is separated from one another by a SC, forming an alternating mosaic pattern. In the auditory epithelium, nectin-1 and nectin-3 are complementarily expressed in HCs and SCs, respectively. During the above-mentioned developmental stages, molecular interactions occur between nectin-1 on HCs and nectin-3 on SCs, where the heterophilic molecular interaction between nectin-1 and -3 is much stronger than the homophilic interactions of nectins between the same type of cells. This biased cell-cell adhesion is responsible for the checkerboard assembly of cells. On the other hand, absence of nectin-3 (Nectin-3 KO) eliminated this bias in cell-cell adhesion, leading to cell rearrangement including attachments between HCs, and an overall disruption of the checkerboard pattern^11,12^.

To unravel the mechanism of this cell rearrangement, we applied the level set-based method to an initial configuration of 12 HCs (orange), 12 SCs (blue), and one pillar cell (gray) aligned, as observed in experiments, at the top and bottom of a rectangular domain ${{\Omega }}=[0,1]\times [0,\frac{5}{8}]$ with periodic boundary conditions both on left and right boundaries, and on top and bottom boundaries (Fig. 5c). We discretized Ω uniformly into *M* = 480 × 300 points and set time step to *δt* = 0.002. We start with interfacial tensions *σ*_SS_ = *σ*_HS_ = *σ*_HH_ = *σ*_SP_ = *σ*_HP_ = 1.0 at E14 stage, changing only the SC-HC tension linearly to *σ*_HS_ = 0.6, which qualitatively follows the change in adhesion strengths measured experimentally in terms of *β*-catenin intensities in auditory epithelium derived from WT mice at E16 stage (see Section *Quantification of Junctional Intensity of Mouse Sensory Epithelium*). Pattern formation at later stages after E16 cannot be explained only by adhesion. Indeed, the change in hair cell morphology from square to circular is thought to be caused by pronounced cytoskeletal dynamics within HCs, leading to increased hair cell stiffness. To express this factor in our model, we evolve the interfacial tensions so that *σ*_SS_ < *σ*_HS_ < *σ*_HH_. Specifically, we impose their linear change reaching *σ*_SS_ = 0.3, *σ*_HS_ = 0.6, *σ*_HH_ = 1.0, *σ*_SP_ = 0.65 and *σ*_HP_ = 1.0 at E18 stage. Figure 5c and Supplementary Movie 4^10^ show that the expected patterns from E14 to E18 stages can be reproduced solely by designing the interfacial tensions. Although the nature of the relationship between interfacial tension and adhesion, contractility, stiffness, internal pressure, etc., is still unknown, our results show the relevant potential of the level set-based approach as an effective tool for investigating this relationship.

Let us further test the proposed method by investigating pattern formation in auditory epithelium of Nectin-3 KO mice, where the strong adhesion between HCs and SCs is inhibited at E16 stage. Based on experimental measurements (see Section *Quantification of Junctional Intensity of Mouse Sensory Epithelium*), this is reflected in the model by setting *σ*_HS_ = 0.9 at E16, while all the remaining values of interfacial tensions are retained from the above WT simulation. The numerical results presented in Fig. 5d and Supplementary Movie 4^10^ show that checkerboard pattern is not formed at E16 and the final pattern is similar to the pattern observed in Nectin-3 KO mice (Fig. 5g). These simulations indicate the importance of transitory formation of checkerboard pattern at the E16 stage for robust attainment of mosaic distribution of HCs and SCs. In Supplementary Note 9, we demonstrate that results of simulation may depend on initial condition but this dependence is eliminated by including fluctuations in the model.

The only publication on mathematical modeling of pattern formation in sensory epithelia that we are aware of, is the recent paper by Cohen et al.^13^. The authors used vertex dynamics model to replicate the checkerboard-like pattern in auditory epithelium, corresponding to Fig. 5a–c (E16). Their main assumptions are global shear motion of sensory HCs and repulsion force between HCs, in addition to tension and volume preservation. However, although repulsive forces between HCs play a central role in their model, there is no real evidence for the existence of such forces. On the other hand, our method, assuming only forces due to interfacial tension and volume preservation, reproduces the final pattern shown in Fig. 5c (E18), which cannot be obtained using vertex dynamics model due to restrictions on available cell shapes. Our work indicates that differential interfacial tension is the main driving force of pattern formation in auditory epithelium. We believe that this result has a substantial impact in developmental biology since a number of researchers expect that intercalation of cells in auditory epithelium requires a convergent extension process whose mechanism is not yet understood^14^.

## Discussion

We have presented a mathematical model together with a computational method for simulating cellular rearrangements occurring in tissue morphogenesis, based on the level set approach. In particular, we adopt an implicit representation of cell-cell junctions and approximate their evolution by a thresholding scheme, which features good compatibility with the level set representation and a solid mathematical background including stability and convergence. We have combined this approach with auction dynamics algorithm to control cell volumes and augmented the numerical scheme with several aspects pertinent to cell biology, most importantly, a localization step which prevents cells from unnatural splitting during their rearrangement.

Because our level set-based approach is simply a numerical implementation of mathematical models founded on free energy minimization, it is therefore necessary to evaluate its pros and cons relative to its known counterparts (e.g., vertex dynamics model), and to specify types of problems, for which it is suitable. We have shown that the proposed level set-based algorithm enjoys the following merits:

1. it is able to accurately express complex geometries of cell-cell junctions and correctly realize cell contact angles, which is indispensable for finding the correct energy minimum and the corresponding dynamics including topology changes such as cell intercalations;
2. it has minimal number of parameters which is essential in using the model as a tool for testing biological hypotheses.

In this respect, the proposed computational method surpasses its counterparts, but at the price of a more complex algorithm and moderate computational cost (see Supplementary Note 6). Thus, it is recommended that one employs vertex dynamics for large-scale problems where detailed understanding of intercalation dynamics is not a priority, while level set-based method is more suitable for relatively small-scale simulations involving frequent topology changes and complex geometry.

A morphogenetic phenomenon, whose understanding essentially relies on these merits, is the formation of cellular patterns in sensory epithelia. Applying our level set-based method to the analysis of its underlying mechanism led us to the discovery that differential interfacial tension plays a decisive role in the formation of sensory epithelia. Indeed, by simulating the epithelial formation based solely on interfacial tensions, we were able to reproduce the evolution of cellular patterns that is observed in experiments. Such finding was not possible until now because established methods either are not able to capture complex curved shapes of cells with largely differing sizes and/or are not sufficiently mathematically accurate to grasp the frequent and delicate topology changes, such as intercalations, that are at the core of the patterning process.

## Methods

### Numerical scheme

We present our algorithm based on the level set approach, starting with the formulation of the mathematical model. We represent an aggregate of cells as a bounded domain ${{\Omega }}\subset {{\mathbb{R}}}^{d}$ (*d* = 2 or 3) partitioned into *N* closed sets ${{{{{{{{\mathcal{C}}}}}}}}}_{1},\ldots ,{{{{{{{{\mathcal{C}}}}}}}}}_{N}$, representing *cells* (see Fig. 1a for the basic notation). It naturally follows that *cell-cell junction* ${\gamma }_{ij}:={{{{{{{{\mathcal{C}}}}}}}}}_{i}\cap {{{{{{{{\mathcal{C}}}}}}}}}_{j}=\partial {{{{{{{{\mathcal{C}}}}}}}}}_{i}\cap \partial {{{{{{{{\mathcal{C}}}}}}}}}_{j}$ is the common boundary of sets ${{{{{{{{\mathcal{C}}}}}}}}}_{i}$ and ${{{{{{{{\mathcal{C}}}}}}}}}_{j}$.

We consider cellular rearrangement as the *L*^2^-gradient flow (see Laux and Otto^7^ and references therein for a precise definition) of the weighted surface energy

2 $$E({{{{{{{{\mathcal{C}}}}}}}}}_{1},\ldots ,{{{{{{{{\mathcal{C}}}}}}}}}_{N})=\mathop{\sum}\limits_{i\ne j}{\sigma }_{ij}\,{{\mbox{Area}}}\,({\gamma }_{ij})$$

constrained by each cell’s prescribed volume ${V}_{\ell }^{0}$ (*ℓ* = 1, 2, …, *N*). Here, Area(*γ*_*ij*_) denotes the area of cell-cell junction *γ*_*ij*_ in a 3d model or the length of the junction in a 2d model, and the weights *σ*_*ij*_ = *σ*_*ji*_ > 0 for *i* ≠ *j* may be related to cell-cell adhesion and/or cell contractility (see Supplementary Note 2 for more). When *i* = *j*, we formally set *σ*_*ij*_ = *σ*_*ii*_ = 0.

In materials science, this problem—considered without volume constraint to begin with—is widely known as the Mullins model for normal grain growth, where ${{{{{{{{\mathcal{C}}}}}}}}}_{i}$ denotes a grain in polycrystalline materials. To realize the least energy, lower semicontinuity of the functional is required, a necessary and sufficient condition for which is the triangle inequality *σ*_*ik*_≤*σ*_*ij*_ + *σ*_*jk*_ for any distinct *i*, *j*, and *k*^15^. The grain boundary *γ*_*ij*_ in the *L*^2^-gradient flow moves with a velocity *μ*_*ij*_*σ*_*ij*_*κ*_*ij*_***η***_*ij*_ where *κ*_*ij*_, *μ*_*ij*_ and ***η***_*ij*_ denote the mean curvature, mobility, and unit normal of *γ*_*ij*_, respectively. Moreover, at triple junction where three grains ${{{{{{{{\mathcal{C}}}}}}}}}_{i}$, ${{{{{{{{\mathcal{C}}}}}}}}}_{j}$, and ${{{{{{{{\mathcal{C}}}}}}}}}_{k}$ meet, the Herring angle condition holds, that is, *σ*_*ij*_***η***_*ij*_ + *σ*_*jk*_***η***_*jk*_ + *σ*_*ik*_***η***_*ik*_ = 0. Thus, grain boundaries in the annealing of pure metals (with equal surface tensions) evolve by mean curvature flow, where triple junctions meet at angles of 120^∘^.

Although the mathematical model is simple, its numerical realization is not at all obvious. Let us first explain the background of the proposed numerical scheme. Earlier works in simulating the above-mentioned grain boundary motion involve front-tracking^16^, which discretizes grain boundaries into finite number of points at which the mean curvature is explicitly calculated to determine its position at next time step. This resembles the vertex dynamics model, in the sense that both approaches evolve vertices based on explicitly calculated quantities. Consequently, its major drawback lies in its inability to handle grain boundaries that cross or have complicated topologies. Proper approximation of the subsequent evolution then requires some form of ad hoc “numerical surgery” which may lack physical justification and can be impractical to implement, particularly in three dimensions. To alleviate this drawback, Merriman et al.^17^ introduced the MBO thresholding scheme for diffusion-generated curvature-dependent motion of multiple junctions, which is based on the level set formulation of Osher and Sethian^18^ for propagating fronts with curvature-dependent speed. This scheme tracks interfaces implicitly by following level sets, facilitating natural handling of topological changes. In recent years, Esedo${\bar{\rm g}}$lu and Otto^3^ extended the MBO method to realize motion of grain boundaries in polycrystalline materials with arbitrary surface tensions. This method inherits the main advantages of the MBO approach: efficiency in the sense of low computational cost; and under a mild condition on the weights *σ*_*ij*_, gradient stability in the sense that in every time step energy (Eq. 2) is decreased.

With the aforementioned advantages, we introduce a level set-based algorithm to simulate cell dynamics in tissue morphogenesis. It is based on the Esedo${\bar{\rm g}}$lu-Otto algorithm but incorporates cell volume constraints and other aspects typical for cells. Following the lines in Esedo${\bar{\rm g}}$lu and Otto^3^, we briefly explain the derivation of the algorithm for the gradient descent of energy (Eq. 2) under the volume constraints

3 $${{{\mathrm{Volume}}}}\,({{{\mathcal{C}}}}_{\ell })={V}_{\ell }^{0},\qquad \ell =1,\ldots ,N.$$

The area (corresponds to “length” in a two-dimensional model) of a cell-cell junction *γ*_*ij*_ can be estimated using the so-called “heat content approximation”, which states that the area of the junction is proportional to the heat that flows from cell ${{{{{{{{\mathcal{C}}}}}}}}}_{j}$ to cell ${{{{{{{{\mathcal{C}}}}}}}}}_{i}$ in a short time *δt*:

4 $${{{{{{\mathrm{Area}}}}}}}\,({\gamma }_{ij})\approx \frac{1}{\sqrt{\delta t}}\int {\chi }_{{{{{{{{{\mathcal{C}}}}}}}}}_{i}}{G}_{\delta t}* {\chi }_{{{{{{{{{\mathcal{C}}}}}}}}}_{j}}\,dx.$$

Here ${G}_{\delta t}(x)={\left(4\pi \delta t\right)}^{-\frac{d}{2}}{e}^{-\frac{| x{| }^{2}}{4\delta t}}$ is the *d*-dimensional Gaussian kernel, where *d* is the spatial dimension of the model, and ${\chi }_{{{{{{{{\mathcal{C}}}}}}}}}$ denotes the characteristic function of a cell region ${{{{{{{\mathcal{C}}}}}}}}$. Hence, multiplying by the weights *σ*_*ij*_ and adding overall junctions, the energy *E* can, with a small error, be replaced by

5 $$E({{{{{{{{\mathcal{C}}}}}}}}}_{1},\ldots ,{{{{{{{{\mathcal{C}}}}}}}}}_{N})\approx {E}_{\delta t}({{{{{{{\boldsymbol{u}}}}}}}}):=\frac{1}{\sqrt{\delta t}}\mathop{\sum }\limits_{i,j=1}^{N}{\sigma }_{ij}\int {u}_{i}{G}_{\delta t}* {u}_{j}\,dx,$$

where we have expressed the cell regions ${{{{{{{{\mathcal{C}}}}}}}}}_{\ell },\ell =1,\ldots ,N$ by a vector-valued function ***u*** = (*u*_1_, …, *u*_*N*_) on Ω, whose components *u*_*ℓ*_(*x*) can take only two values: 1 if the point *x* belongs to ${{{{{{{{\mathcal{C}}}}}}}}}_{\ell }$, or 0 if it does not. It was shown^3^ that *E*_*δt*_ is a correct approximation of the original energy *E* in the sense that it Γ-converges to *E* when *δt* → 0.

Due to the condition on cell volumes, function ***u*** is constrained to the set

6 $${{{{{{{\mathcal{B}}}}}}}}:=\left\{{{{{{{{\boldsymbol{u}}}}}}}}\in {\{0,1\}}^{N}:\mathop{\sum }\limits_{j=1}^{N}{u}_{j}(x)=1\ \,{{\mbox{a.e.}}}\,\,x\in {{\Omega }}\ \,{{\mbox{and}}}\,\ \int_{{{\Omega }}}{u}_{\ell }={V}_{\ell }^{0},\ell =1,\ldots ,N\right\},$$

which is not convex. This poses a difficulty in the minimization problem, but it can be shown in a similar fashion to Esedo${\bar{\rm g}}$lu and Otto^3^ that the minimum of *E*_*δt*_ over ${{{{{{{\mathcal{B}}}}}}}}$ coincides with the minimum over the convex set ${{{{{{{\mathcal{K}}}}}}}}$ obtained from ${{{{{{{\mathcal{B}}}}}}}}$ by relaxation, i.e., by allowing the components of ***u*** to take any value between 0 and 1:

7 $${{{{{{{\mathcal{K}}}}}}}}:=\left\{{{{{{{{\boldsymbol{u}}}}}}}}\in {[0,1]}^{N}:\mathop{\sum }\limits_{j=1}^{N}{u}_{j}(x)=1\ \,{{\mbox{a.e.}}}\,\,x\in {{\Omega }}\ \,{{\mbox{and}}}\,\ \int_{{{\Omega }}}{u}_{\ell }={V}_{\ell }^{0},\ell =1,\ldots ,N\right\}.$$

The approximate energy *E*_*δt*_ is still nonlinear, so to devise a simple minimization scheme, we adopt an iterative process by virtue of Lemma 5.2 in Esedo${\bar{\rm g}}$lu and Otto^3^ as follows. Given an approximation ***u***^*k*^ of the minimizer of *E*_*δt*_ in ${{{{{{{\mathcal{K}}}}}}}}$, we compute the next best approximation ***u***^*k*+1^ by linearizing energy *E*_*δt*_(***u***) around ***u***^*k*^ and defining ***u***^*k*+1^ to be the minimizer of the linearized energy over ${{{{{{{\mathcal{K}}}}}}}}$:

8 $${{{{{{{{\boldsymbol{u}}}}}}}}}^{k+1}=\mathop{{{{{{\rm{arg}}}}}}\,{{{{{\rm{max}}}}}}}\limits_{{{{{{{{\boldsymbol{u}}}}}}}}\in {{{{{{{\mathcal{K}}}}}}}}}{{{{{{{{\mathcal{L}}}}}}}}}_{{E}_{\delta t}}({{{{{{{\boldsymbol{u}}}}}}}};{{{{{{{{\boldsymbol{u}}}}}}}}}^{k}).$$

Here ${{{{{{{{\mathcal{L}}}}}}}}}_{{E}_{\delta t}}$ is the linearized energy given by

9 $${{{{{{{{\mathcal{L}}}}}}}}}_{{E}_{\delta t}}({{{{{{{\boldsymbol{u}}}}}}}};{{{{{{{{\boldsymbol{u}}}}}}}}}^{k})=\frac{2}{\sqrt{\delta t}}\mathop{\sum }\limits_{i=1}^{N}\int {\varphi }_{i}^{k}{u}_{i}\,dx,\quad {\varphi }_{i}^{k}:=\mathop{\sum }\limits_{j=1}^{N}{\sigma }_{ij}{G}_{\delta t}* {u}_{j}^{k}.$$

In the main algorithm below, we use function ${\psi }_{i}^{k}=1-{\varphi }_{i}^{k}$ instead of ${\varphi }_{i}^{k}$ for the purpose of reformulating the minimization problem into a maximization one. It was proved^3,7^ that in the absence of volume constraints the sequence {***u***^*k*^} decreases the approximate energy *E*_*δt*_ with increasing step number *k* and correctly approximates the *L*^2^-gradient flow of the original energy *E* in the limit *δt* → 0.

In the case when there is no volume constraint, minimization (Eq. 8) becomes a problem of minimizing a linear function over a simplex set ${{{{{{{\mathcal{K}}}}}}}}$. Thus the solution is obtained immediately as

10 $${u}_{i}^{k+1}(x)=\left\{\begin{array}{ll}1\quad &\,{{\mbox{if}}}\,\ {\varphi }_{i}^{k}(x)={\min }_{j}{\varphi }_{j}^{k}(x)\\ 0\quad &\,{{\mbox{otherwise}}}\,\end{array}\right.$$

which leads to a very simple thresholding scheme. However, when the set ${{{{{{{\mathcal{K}}}}}}}}$ includes volume constraints, the solution of the minimization (Eq. 8) involves unknown Lagrange multipliers *λ*_*ij*_:

11 $${u}_{i}^{k+1}(x)=\left\{\begin{array}{ll}1\quad &\,{{\mbox{if}}}\,\ {\varphi }_{i}^{k}(x)={\min }_{j}({\varphi }_{j}^{k}(x)+{\lambda }_{ij})\\ 0\quad &\,{{\mbox{otherwise .}}}\,\end{array}\right.$$

Direct computation of the Lagrange multipliers for more than three cells is complicated and can be avoided by the application of auction algorithm^8^. The idea is to discretize the domain Ω into a uniform grid of points ${\omega }_{M}={\{{x}_{m}\}}_{m = 1}^{M}\subset {{\Omega }}$ and assign cell membership to each point of *ω*_*M*_ by simulating an auction, so that in the end each cell ${{{{{{{{\mathcal{C}}}}}}}}}_{\ell }$ contains ${v}_{\ell }^{0}$ points. Here, the number of grid points ${v}_{\ell }^{0}$ corresponds to the volume of the cell ${{{{{{{{\mathcal{C}}}}}}}}}_{\ell }$ in the sense of ${v}_{\ell }^{0}/M\approx {V}_{\ell }^{0}/| {{\Omega }}|$. It is natural to take the grid nodes {*x*_*m*_} identical to the grid nodes of the mesh used to numerically realize the convolutions in (Eq. 9). The starting point of the auction dynamics algorithm is the configuration of the cells obtained by the gradient flow of energy without any volume constraint which is determined by the functions ${\varphi }_{i}^{k}$ above, or by the functions ${\psi }_{i}^{k}$ in the Algorithm below. In this configuration, some cells expand and some deflate with respect to their original volume. The algorithm then starts with all grid points unassigned and in arbitrary order takes the yet unassigned points and assigns them to their most preferred cell, where the extent of preference is in the beginning determined solely by the unconstrained configuration but later have to be adjusted via two new variables, namely the grid point’s bid and the cell’s price. This is because the “popular” cells, i.e., those which tend to expand in the unconstrained motion, become full in the sense that they reach the upper limit of points that can be accepted in them due to the volume constraint, and thus either some points have to be kicked out of the cell or the current point has to be assigned to its second favorite cell. The design of bids and prices that direct the unassigning and accepting of points resembles an auction performed by the grid points on the cells, leading to the naming of the algorithm. It is proved that the kicking out and accepting process finishes in a finite number of cycles and leads to the exact solution (at the discrete level of grid points) of the volume-constrained minimization problem. We refer to the main Algorithm below for precise description of the steps and to Jacobs et al.^8^ for a more concise exposition and rigorous proofs.

The Esedo${\bar{\rm g}}$lu-Otto scheme is simple and efficient but there are some issues that need to be tackled, in particular, the phenomena of wetting and nucleation^3^. Failure to satisfy the *σ*-triangle inequality condition leads to wetting, where a new cell ${{{{{{{{\mathcal{C}}}}}}}}}_{n}$ suddenly appears along an unrelated cell-cell junction *γ*_*ij*_. Moreover, even when *σ*-triangle inequality is satisfied, a new cell may still get nucleated at a tricellular junction. For evolutions computed with auction dynamics, such wetting and nucleation will force a cell to split into two or more disjoint parts, some of which transfer to the wetting or nucleation regions. It is important to address this issue since such cell splitting phenomena do not occur during cellular rearrangements; yet it is possible that *σ*_*ij*_’s may not necessarily satisfy the triangle inequality condition in real tissues (see Supplementary Note 3 for more detailed exposition). To this end, we modify the auction algorithm by incorporating a topological constraint, so as to preserve cell connectivity. This makes sense physically, since individual cells only move in response to their local surroundings, i.e., to their neighboring cells. Hence, when we establish cell membership, we only allow local bidding processes in the auction, as shown in the following main algorithm (see Fig. 1b for illustrations).

#### Algorithm 1

(for numerical approximation of the *L*^2^-gradient flow of energy (Eq. 2) with preservation of cell volumes and connectivity)

***Notation:*** Denote by ${\chi }_{{{{{{{{\mathcal{C}}}}}}}}}$ the characteristic function of a set ${{{{{{{\mathcal{C}}}}}}}}$. For given grid points ${\omega }_{M}={\{{x}_{m}\}}_{m = 1}^{M}\subset {{\Omega }}$ and a cell region ${{{{{{{\mathcal{C}}}}}}}}$, define the number of grid points in ${{{{{{{\mathcal{C}}}}}}}}$ by $\left|{{{{{{{\mathcal{C}}}}}}}}\right|:=\#\{m:{x}_{m}\in {{{{{{{\mathcal{C}}}}}}}}\}$.

***Initialization:*** Split the time interval [0, *T*] into *K* subintervals of equal length *δt* = *T*/*K*. Discretize the computational domain Ω into a uniform finite grid ${\omega }_{M}={\{{x}_{m}\}}_{m=1}^{M}\subset {{\Omega }}$. Prescribe initial cell regions ${\left\{{{{{{{{{\mathcal{C}}}}}}}}}_{n}^{0}\right\}}_{n=1}^{N}$ by assigning each discrete point *x*_*m*_ ∈ *ω*_*M*_ to a cell region. For each *n* = 1, …, *N*, record ${v}_{n}^{0}=| {{{{{{{{\mathcal{C}}}}}}}}}_{n}^{0}|$, the number of grid points in ${{{{{{{{\mathcal{C}}}}}}}}}_{n}^{0}$. Set the weights ${\{{\sigma }_{ij}^{k}\}}_{i,j = 1,\ldots ,N,k = 0,\ldots ,K-1}$ (here index *k* refers to time) and the initialization parameter 0 < *ε* ≪ 1 for the auction algorithm.

For each time step *k* = 0, 1, …, *K* − 1 perform the following steps, in order to determine the numerical cell regions ${\left\{{{{{{{{{\mathcal{C}}}}}}}}}_{n}^{k+1}\right\}}_{n = 1}^{N}$ at time *t* = (*k* + 1)*δt*:

1. *Solving heat equation*. For each *x*_*m*_ ∈ *ω*_*M*_ compute
  12 $${\psi }_{i}^{k}({x}_{m}):=1-\mathop{\sum }\limits_{j=1}^{N}{\sigma }_{ij}^{k}\left({G}_{\delta t}* {\chi }_{{{{{{{{{\mathcal{C}}}}}}}}}_{j}^{k}}\right)({x}_{m}),$$
  where ${G}_{\delta t}(x)={\left(4\pi \delta t\right)}^{-\frac{d}{2}}{e}^{-\frac{| x{| }^{2}}{4\delta t}}$ is the *d*-dimensional Gaussian kernel.
2. *Localized auction dynamics*. Initialize prices *p*_*n*_ = 0 and cell regions ${{{{{{{{\mathcal{C}}}}}}}}}_{n}^{k+1}={{\emptyset}}$ (*n* = 1, …, *N*). Until all points in *ω*_*M*_ are assigned, do:
  - Find a point *x*_*m*_ ∈ *ω*_*M*_ which is not assigned.
  - Let *i* be the index of the cell to which *x*_*m*_ belonged at step *k*.
  - Find the set of the indices of neighboring cells of ${{{{{{{{\mathcal{C}}}}}}}}}_{i}^{k}$: ${{{{{{{{\mathcal{N}}}}}}}}}_{{x}_{m}}:=\{j:\partial {{{{{{{{\mathcal{C}}}}}}}}}_{i}^{k}\cap \partial {{{{{{{{\mathcal{C}}}}}}}}}_{j}^{k}\;\ne\; {{\emptyset}}\}$.
  - Determine
    13 $${i}^{* }=\mathop{{{{{{\rm{arg}}}}}}\,{{{{{\rm{max}}}}}}}\limits_{i\in {{{{{{{{\mathcal{N}}}}}}}}}_{{x}_{m}}}\left({\psi }_{i}^{k}({x}_{m})-{p}_{i}\right),\quad {i}^{\sharp }=\mathop{{{{{{\rm{arg}}}}}}\,{{{{{\rm{max}}}}}}}\limits_{i\in {{{{{{{{\mathcal{N}}}}}}}}}_{{x}_{m}}\backslash \{{i}^{* }\}}\,\,\left({\psi }_{i}^{k}({x}_{m})-{p}_{i}\right).$$
  - If $$| {{{{{{{{\mathcal{C}}}}}}}}}_{{i}^{* }}^{k+1}| ={v}_{{i}^{* }}^{0}$$,
    • find *l* such that ${x}_{l}={\arg\min}_{x\in {{{{{{{{\mathcal{C}}}}}}}}}_{{i}^{* }}^{k+1}}b(x)$, and
    • unassign *x*_*l*_ from $${{{{{{{{\mathcal{C}}}}}}}}}_{{i}^{* }}^{k+1}$$.
  - Assign *x*_*m*_ to $${{{{{{{{\mathcal{C}}}}}}}}}_{{i}^{* }}^{k+1}$$.
  - Calculate the bid
    14 $$b({x}_{m})={p}_{{i}^{* }}+\varepsilon +\left({\psi }_{{i}^{* }}^{k}({x}_{m})-{p}_{{i}^{* }}\right)-\left({\psi }_{{i}^{\sharp }}^{k}({x}_{m})-{p}_{{i}^{\sharp }}\right).$$
  - Update the price ${p}_{{i}^{* }}=\mathop{\min }_{x\in {{{{{{{{\mathcal{C}}}}}}}}}_{{i}^{* }}^{k+1}}b(x)$.

It can be shown that thanks to the positive value of *ε*, the second auction dynamics step finishes in finitely many steps, yielding a partition of *ω*_*M*_ into cell regions, such that each cell region has the prescribed number of grid points^8^. Characteristic functions of this partition are fed into the first step of the algorithm and the algorithm repeats until the final time is reached. The bid function *b* appears for the first time in step 2(e) of the algorithm without being previously initialized but this definition is consistent since the bid has meaning only for grid points that are already assigned to a cell region. The bid expresses the extent to which the grid point “wants to be a member” of the cell region which it currently belongs to, and its role is to resolve conflicts among grid points that are trying to become members of the same, already full cell region. Supplementary Movie 5^10^ shows an animation of the auction dynamics algorithm for one time step of rearrangement in a simple 3-cell aggregate, tracking current cell price, node bid and nodal assignments.

We now briefly comment on the parameters related to the numerical implementation. The discretization parameters are the number *M* of discrete points in the computational domain Ω and the time step size *δt*. The convolutions (Eq. 12) in each step are efficiently computed on rectangular grids using fast Fourier transform (FFT) algorithm with a complexity of $O(M\log M)$ operations^3^. The time step can be changed throughout the computation but we emphasize that there are restrictions on the relative size of the spatial and temporal grids in order to obtain reasonable results; namely, an excessively small time step relative to the space grid size leads to incorrect stagnation of moving level sets. A common practice is to take *δt* proportional to the first power of the spatial grid size *δx*^19^. Moreover, the parameter *ε* of the auction algorithm is taken as a small positive value and has the role of preventing a “price war” infinite loop, where the prices *p*_*i*_ get stuck at a certain value. Too small *ε* may result in an increase in computational time, while a large value may lead to deviations from the prescribed cell volumes. An idea of *ε*-scaling introduced in Jacobs et al.^8^ consists in starting with a relatively large *ε* and repeating the auction with smaller and smaller values of *ε*, which not only eliminates the influence of this parameter but also improves both computational time and accuracy. The complexity of the auction step for a fixed *ε* is $O(Nv(\log v+N)C/\varepsilon )$, where $v=\mathop{\max}\limits_{i}{v}_{i}^{0}$ and $C=\mathop{\max}\limits_{i,x}{\psi }_{i}^{k}(x)$^8^. In summary, except for the unavoidable discretization parameters *M* and *δt*, the output of the algorithm depends solely on the model’s physically meaningful parameters *σ*_*ij*_. The meaning and choice of these parameters are discussed in Supplementary Note 2.

Regarding the choice of boundary conditions we remark that the Esedo${\bar{\rm g}}$lu-Otto scheme is originally formulated in the whole space ${{\mathbb{R}}}^{d}$ and thus numerical implementation on a bounded domain requires additional modifications. The simplest approach, which is usually compatible with biological settings, is to adopt a rectangular computational domain and apply Fourier transform to solve the convolutions (Eq. 12). This naturally leads to periodic boundary condition for the evolution. Application of Fourier transform also allows for boundary conditions of Neumann type but other types of boundary conditions may require nontrivial adjustments and conceding the effective FFT method in favor of more general but more costly algorithms.

Next, we summarize basic mathematical properties of the algorithm, i.e., its stability and convergence. Firstly, for the original algorithm without volume constraint, Esedo${\bar{\rm g}}$lu and Otto^3^ showed that it is unconditionally gradient stable: for any choice of the time step *δt*, it dissipates in every time step the approximate energy (Eq. 5) (which Γ-converges to energy (Eq. 2)) under the sufficient condition that the surface tension matrix ${\{{\sigma }_{ij}\}}_{i,j = 1}^{N}$ is conditionally negative semi-definite:

15 $$\mathop{\sum }\limits_{i,j=1}^{N}{\sigma }_{ij}{\xi }_{i}{\xi }_{j}\le 0\qquad \,{{\mbox{for any}}}\,\ ({\xi }_{1},\ldots {\xi }_{N})\in {{\mathbb{R}}}^{N}\ \ \,{{\mbox{such that}}}\,\quad \mathop{\sum }\limits_{i=1}^{N}{\xi }_{i}=0.$$

This condition is often satisfied in materials science but there is no guarantee that it will hold in biological settings, e.g., cell-cell adhesiveness strengths in OE measured in terms of its *β*-catenin intensity values^6^. In such a case, it is possible to devise a slightly more complex version of the algorithm that guarantees gradient stability solely under the *σ*-triangle inequality condition (we refer to Section 5.4 of Esedo${\bar{\rm g}}$lu and Otto^3^ for details). The convergence of the algorithm to the weak solution of the *L*^2^-gradient flow of energy (Eq. 2) has been proved by Laux and Otto^7^. We note that due to the fundamental idea of the algorithm to propagate interfaces over a fixed grid and due to the stagnation phenomenon mentioned above, the order of convergence is restricted to at most 1 in both time and space, while the order near multiple junctions turns out to be only $\frac{1}{2}$ in time.

The nontrivial difficulty in the analysis of the volume-preserving combined algorithm lies in the fact that the auction algorithm is in essence space-discrete, while all existing proofs deal with space-continuous problems. Moreover, in our scheme we localize the auction step which makes the analysis even more involved. However, the stability of the volume-constrained problem in the space-continuous setting (formulated using Lagrange multipliers for each cell’s volume) can be proved in the same way and under the same assumptions as in the unconstrained case (see Xu et al.^20^ for the idea of the proof), while convergence has been established in Laux and Swartz^21^. This, together with the known convergence of the auction algorithm (at the spatially discretized level, the optimal solution can be achieved precisely) supports the expectation for the correct behavior of the combined scheme, which still remains to be precisely proved. Since a rigorous proof is beyond the scope of the paper, we have included a series of numerical tests confirming the correct behavior of our scheme. An exhaustive account on the results of these numerical tests is provided in Supplementary Note 4 with the following conclusions. The scheme is of order one in time away from junctions and the order of convergence falls to around 0.5 when triple junctions are involved. This was tested also on a configuration involving topology change. However, this convergence property holds true only for time steps larger than a certain threshold depending on the spatial grid—if the time step *δt* is several times smaller than the grid size *δx* then interfaces stagnate and the scheme fails to converge. Further, the algorithm was tested on a three-phase configuration with triple junctions leading to an anisotropic double bubble and it was confirmed that its output closely follows an accurate front-tracking approximation of the evolution, with error decreasing with refinement of discretization. The same holds true for the stationary solution of double bubble evolution.

### Immunofluorescence microscopy

To prepare whole-mount samples of OE from embryos and P1 mice, the OE was dissected out and fixed with 4% paraformaldehyde (PFA) in Hank’s balanced salt solution (HBSS) containing 1 mM Ca^2+^ and Mg^2+^ at 4 °C for 1 h or overnight. The olfactory mucosa was dissected out and fixed at 4 °C for 1 h. They were decapitated and post-fixed for 3 h at 4 °C in the fixative. To avoid variations in developmental growth rates between the regions of interest, the observation of the OE was restricted to the posterior ventral part of the nasal septum.

To visualize cells, the cells were fixed in 4% PFA in HBSS for 10–15 min at room temperature. After treatment with 0.25% Triton X-100 in TBST (TBS with 0.005% Tween-20) for 5 min, the cells were blocked with 5% skim milk in TBST and exposed for 2 h to primary antibodies in 5% skim milk in TBST.

The Abs used were: mouse anti-mouse *β*-catenin (Clone 5H10; Invitrogen) and rat anti-mouse nectin-1 monoclonal Ab (mAb) (clone 48-12; MBL), rat anti-mouse nectin-3 mAb (clone 103-A1; MBL), rat anti-E-cadherin mAb (clone ECCD2, Takara), and mouse anti-N-cadherin mAb (clone 32, BD Transduction Laboratories). Primary Abs were visualized with goat fluorochrome-conjugated secondary Abs. The fluorochromes used were Alexa Fluor 488 and 555 (Invitrogen). Images of a whole-mount of OE or cell culture were obtained using a confocal microscope (LSM700; Carl Zeiss) equipped with a 40 × NA 1.2 lens using ZEN software (Carl Zeiss).

### Quantification of junctional intensity of mouse sensory epithelium

Measurements of *β*-catenin intensity of cell-cell junctions in the mouse olfactory and auditory epithelium were performed by quantifying the pictures of specimens using ZEN software (Carl Zeiss). Quantifications were performed for normalized fluorescence intensities, and the average was calculated. *α*N-catenin KO mice and nectin-3 KO mice were generated as previously described^22,23^. The animal experiments were approved by the Institutional Animal Care and Use Committee and carried out according to the Kobe University Animal Experimental Regulations.

### Statistics and reproducibility

Data were collected from three individuals and were quantified using five fields in each individual. A *p* value of < 0.05 was considered statistically significant.

### Reporting summary

Further information on research design is available in the Nature Research Reporting Summary linked to this article.

## Supplementary information

Peer Review File

Supplementary Information

Reporting Summary

## Data Availability

All data generated or analyzed during this study are included in this published article (and its supplementary information files) or are fully accessible in the cited previous work. Data used to generate plots, together with supplementary movies are available on Figshare (10.6084/m9.figshare.18070421^24^, 10.6084/m9.figshare.18070424^10^). Accession numbers for cDNAs are listed as below: mouse nectin-1 gene, AF297665.1; mouse nectin-3 gene, NM_021495.4; mouse cadherin-1 gene, NM_009864.3; mouse cadherin-2 gene, NM_007664.5.

## Code availability

The code implementing the proposed algorithm to obtain all level set-based simulations is fully accessible on Zenodo (10.5281/zenodo.5834687).
