# Supplementary material for: A numerical algorithm for modeling cellular rearrangements in tissue morphogenesis
Source: Commun Biol. 2022 Mar 18;5:239. doi: 10.1038/s42003-022-03174-6 (PMC8933555; doi:10.1038/s42003-022-03174-6)
Supplement: Supplementary file 2 — Supplementary Information [file 42003_2022_3174_MOESM2_ESM.pdf]

Supplementary Information

A numerical algorithm for modeling cellular rearrangements in tissue morphogenesis

Rhudaina Z. Mohammad[1,2], Hideki Murakawa[3], Karel Svadlenka[1,4,*], and Hideru Togashi[5,6]

[1]*Department of Mathematics, Graduate School of Science, Kyoto University, Kyoto, Japan*
[2]*Institute of Mathematics, College of Science, University of the Philippines Diliman, Quezon City, Philippines*
[3]*Applied Mathematics and Informatics Course, Faculty of Advanced Science and Technology, Ryukoku University, Otsu, Japan*
[4]*Mathematical Institute, Czech Academy of Sciences, Prague, Czech Republic*
[5]*JST PRESTO (Precursory Research for Embryonic Science and Technology), Kobe, Japan*
[6]*Department of Biochemistry and Molecular Biology, Kobe University Graduate School of Medicine, Kobe, Japan*

[*]Corresponding author's email: `karel@math.kyoto-u.ac.jp`

Supplementary Note 1. Overview of Numerical Methods

Morphogenetic phenomena can be investigated at various scales ranging from molecular up to continuum level; see, e.g. Armstrong et al.[1], Carrillo et al.[2] for a successful reproduction of cellular patterns formed due to cell-cell adhesion and other factors at the continuum level. In this work, our objective is to understand the mechanisms of interactions among cells on the microscopic level, thereby, requiring us to precisely resolve evolving shapes of individual cells.

Supplementary Figure 1: Major numerical models for tissue morphogenesis.

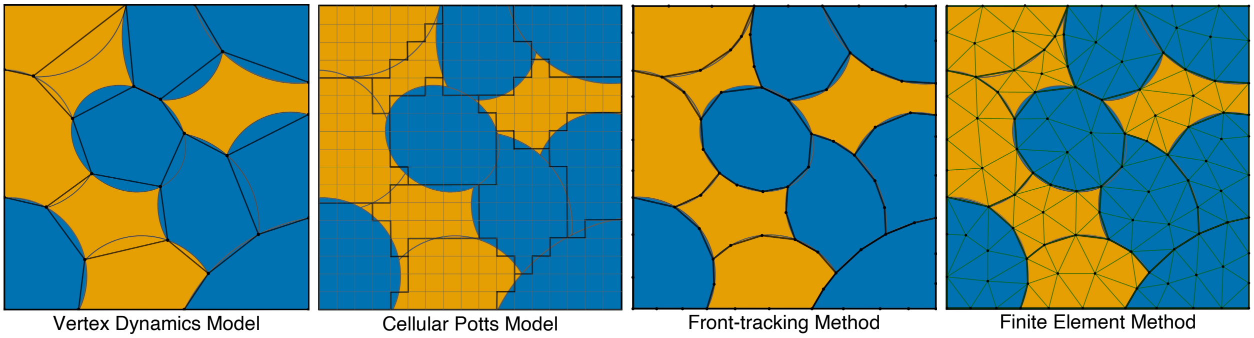

An aggregate of orange and blue cells geometrically represented according to vertex dynamics model, cellular Potts model, front-tracking, and finite element method.

Numerical methods for free energy minimization at microscopic scale include, among others, vertex dynamics model, cellular Potts model, front-tracking, and finite element methods (see Supplementary Figure 1). The main difference of these approaches lies in how cells are geometrically represented. Vertex dynamics model[3,4] represents cells as polygons where mechanical forces are applied to the vertices. In this model, polygonal vertices migrate so as to decrease an energy potential, which includes a penalty term to enforce each cell's preferred volume. Such geometric representation, however, cannot precisely approximate cell-cell junctions of complex shapes with nonzero curvature and tissues composed of cells with largely differing sizes. To resolve this problem, Ishimoto and Morishita[5] proposed the "bubbly vertex dynamics" where curvature of junctions are included as a parameter of the cell geometry. Cellular Potts model[6], on the other hand, discretizes the continuous cell aggregate configuration onto a fixed regular lattice; thereby, representing each cell as a number of grid points. At each grid point, one calculates how the energy (based on cell-cell adhesion, cell incompressibility, and chemotaxis) changes as the grid point transitions to a randomly selected neighbor. If

this elicits a decrease in energy, the grid point is allowed to transition to its neighbor. As a result, when a cell moves, it may lose or gain some grid points on the lattice. Due to its local nature, this approach does not have the potential to accurately deal with topology changes triggered by a global energy balance. Another approach based on the finite element method[7,8,9] partitions polygonal cells into a finite number of triangular elements to solve mechanical equations characterizing tissue dynamics. In the two-dimensional setting, each cell-cell junction is represented as a piecewise linear curve and interfacial tensions are modeled using constant force on point masses along each cell-cell junction. In this sense, this formulation acts like the vertex dynamics model. In a similar vein, front-tracking method approximates junctions by discrete points, which are moved by the given evolution law. The well-known Brakke's Surface Evolver[10], for example, can simulate various types of interface evolution, including multiphase evolutions in 3 dimensions. Applications to complex cellular rearrangements including the analysis of its performance with respect to frequent topology changes, however, are yet to be addressed.

Supplementary Note 2. Model Parameters

Here we elaborate on physically relevant parameters that are required or allowed by the model introduced in Methods. Apart from parameters M, δt and ε used in the numerical implementation of the model, the only parameters of the model itself are the weights σ_{ij} – a clear advantage over other similar models. Vertex dynamics model, for example, requires, in addition to these weights, parameters such as elastic stiffness coefficients for the volume and perimeter, minimal distance between vertices to initiate T1 transformation, etc. Consequently, our model allows us to focus on the role of the target force represented by the parameters σ_{ij} without having to analyze the impact of other effects. This is essential as such analysis often presents an infeasible task.

The choice of values for σ_{ij}'s depends on the purpose of the modeling, and usually requires novel ideas and insights into the modeled phenomenon. Moreover, these values act as a doorway through which experimental measurements can be reflected in the model, in a quantitative manner, and thus their choice is also closely tied to the design of experiments. In this sense, it is impossible to devise a general recipe, and we will only give some examples of possible choices for σ_{ij}'s. There are two competing approaches to the design of σ_{ij}, which, we believe, are peacefully united in our model: Steinberg's differential adhesion hypothesis[11,12] and Brodland's differential interfacial tension hypothesis[13]. Steinberg[11] based his theory on the observation of similarities between liquid sorting and cell sorting, and postulated that cellular rearrangements can be in certain cases explained by the tendency of the cells to maximize their intermolecular adhesion. Evidences speaking for the correctness of the hypothesis were provided by several experiments, the prominent one being the establishment of a hierarchical sequence of segregation among several different tissues. Later, Brodland[13] pointed out that adhesion acts in the opposite direction than interfacial tension, and formulated a more complete and precise hypothesis, giving it a new name. Discussions in the biological modeling community on the implications of these and other hypotheses have been conducted for several decades[14,7]. Our purpose here is not necessarily to probe into these discussions; but rather to present a general model that can encompass most of the approaches proposed up to date. For example, the model can take the weights σ_{ij} to be the cortical tensions of the cell membrane[15] (in which case there is a direct correspondence with the physical meaning of these parameters), or the weights may reflect the adhesion energy per area[16,17] (in which case σ_{ij}'s do not have the meaning of adhesion energy but depend on that energy in a suitable manner that has to be determined as a part of the particular model), or a combination of both. In the main text (Section 2.3), we give a specific example of the design of weights σ_{ij} expressing adhesion energy quantified through experimentally measurable quantity of β-catenin intensity. We recognize that indeed, a precise quantification of the relation between measured β-catenin intensities and magnitude of adhesion strength of the corresponding interfacial tension is still a challenging task. Although several techniques have been proposed to quantify the strength of cadherin–dependent cell–cell adhesion, e.g., flow chamber assay, atomic force microscopy, and dual pipette assay; application of these techniques to intact tissue does not seem feasible. Here, we reiterate that we define cell-cell adhesion strength based on the measured value of β-catenin intensity from intact tissue, because β-catenin intensity is known to be correlated with adhesion strength.

Next, we bring attention to the fact that weights σ_{ij} in the algorithm can be time-dependent, which is expressed by the index k in equation (12). This is essential, as almost all morphogenetic phenomena are driven by temporally changing forces. Moreover, this time-dependence turns our model from a mere energy minimizing gradient descent system into an out-of-equilibrium one, as expected for a model of a living system.

Lastly, the minimal set of parameters, i.e., the weights σ_{ij}, can be augmented by new parameters according to necessity, say, to express various additional aspects of the target biological phenomenon. One example of such additional parameters are the mobilities μ_{ij} mentioned in the beginning of Methods. In fact, the algorithm as presented in the main text (Section 2.1) advances each junction γ_{ij} with the mobility $\mu_{ij} = 1/\sigma_{ij}$. If one wishes to prescribe mobilities in a different way, it is possible to modify the algorithm by introducing the so-called retardation terms[18, 19]. Another optional set of parameters $\{a_i^k, b_i^k\}_{i=1}^N$ is related to the lower and upper bounds controlling the volumes of individual cell regions, thus allowing for certain cell compressibility, in the sense that we look for the energy minimizing configuration such that the discrete cell volumes v_i^k (i.e., the number of grid points in cell $\mathcal{C}_i$ at a given time t_k) satisfy

$$a_i^k \leq v_i^k \leq b_i^k, \qquad i = 1, \ldots, N. \tag{S.1}$$

It is possible to modify the auction algorithm to extend it to this type of constraint. The implementation of the upper bound does not substantially change the algorithm but the lower bound requires running a reverse auction where cell regions bid on points[20]. One can also incorporate random effects in the algorithm – either by randomly changing cell volumes[20] or by adding a suitable noise to the weights σ_{ij} (see Supplementary Note 9).

Supplementary Note 3. Topological Singularities

In this note, we show that our localization scheme resolves the wetting and nucleation problem of the original Esedoḡlu-Otto algorithm.

Consider a wetting case for a 4-cell aggregate of three types: 2 blue, 1 orange, and 1 gray cell (see Supplementary Figure 2), with $\sigma_{\mathrm{BB}} = 1.5$, $\sigma_{\mathrm{BG}} = 0.5$, and $\sigma_{\mathrm{BO}} = \sigma_{\mathrm{OO}} = \sigma_{\mathrm{OG}} = \sigma_{\mathrm{GG}} = 1.0$. Here, $\mathrm{B}, \mathrm{O}, \mathrm{G}$ denote the cells of type blue, orange and gray, respectively, so that, for example, σ_{BO} means the interfacial energy weight for the junction between blue and orange cell. Note that this violates the triangle inequality, since $\sigma_{\mathrm{B_1G}} + \sigma_{\mathrm{B_2G}} = 1 < 1.5 = \sigma_{\mathrm{B_1B_2}}$. We evolve the initial configuration using three different algorithms: the original Esedoḡlu-Otto scheme[18] (without volume constraint), the same scheme with standard auction dynamics[20] to preserve volume, and finally with our proposed scheme employing a localized auction dynamics to preserve both volume and cell connectivity. Here, we discretize domain $\Omega = [0,1] \times [0,1]$ uniformly into $M = 500 \times 500$ points, prescribe periodic conditions on its boundary, and set time step $\delta t = 0.0005$. Results of the simulation are shown in Supplementary Figure 2 and Supplementary Movie 6.

We observe that for the Esedoḡlu-Otto scheme, a new gray cell grows at the BB-junctions – wetting occurs. When implemented with the usual auction dynamics, we see that the gray cell splits and some of its parts appear in the BB-junction. In these cases, optimization (see Methods, equation (8)) is taken over all possible cells, and thus σ-triangle inequality becomes important to rule out wetting. However, with the localized auction dynamics, cell splitting due to wetting is avoided. This is because, for points in the neighborhood of the BB-junction, maximization (see Methods, equation (13)) is localized and only taken over the two blue cells. Hence, irregardless of whether σ-triangle inequality holds, wetting does not occur with localized auction dynamics.

Next, we consider a nucleation case for a 4-cell aggregate of two types: 3 blue and 1 orange cell (see Supplementary Figure 3) under the same conditions on the domain and time step size as in the first simulation. With $\sigma_{\mathrm{BB}} = \sigma_{\mathrm{OO}} = 1.0$ and $\sigma_{\mathrm{BO}} = 0.53$, we evolve the initial configuration using the same numerical schemes as above. Note that since the σ_{ij}'s satisfy the triangle inequality condition, wetting cannot take place. However, Supplementary Figure 3 and Supplementary Movie 7 show that in the

evolution computed by Esedoḡlu-Otto algorithm alone, orange cells grow in the vicinity of the blue tricellular junction – an unnatural cell dynamics. This phenomenon persists even when auction algorithm is incorporated, but is completely eliminated upon introducing the localization.

Supplementary Figure 2: Wetting phenomenon in the numerical implementation of level set-based methods

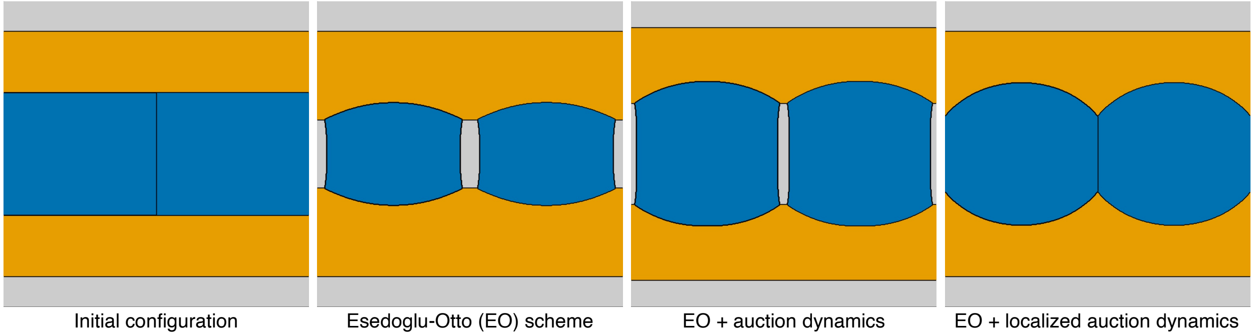

Initial 4-cell configuration and snapshots of its evolution under a wetting condition due to violation of σ-triangle inequality at $t = 50\delta t$ using Esedoḡlu-Otto scheme (EO); EO scheme with auction dynamics algorithm; and EO scheme with localized auction dynamics.

Supplementary Figure 3: Nucleation phenomenon in the numerical implementation of level set-based methods

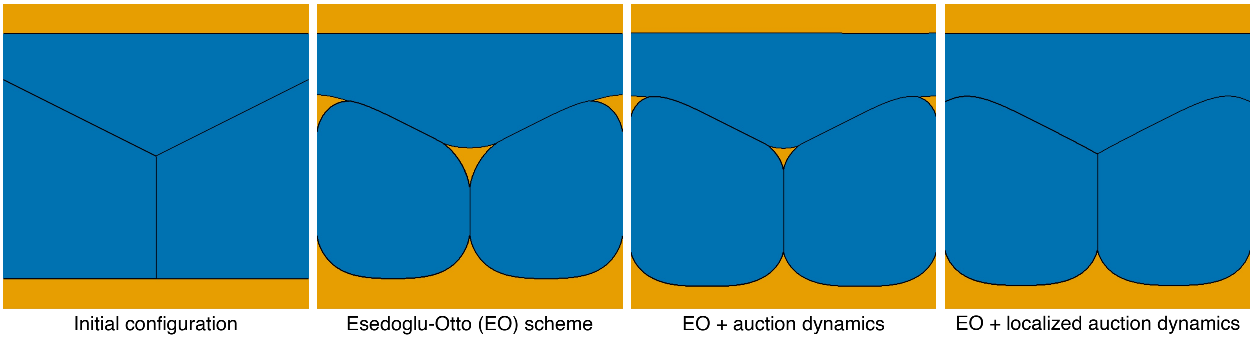

Initial 4-cell configuration and snapshots of its evolution at $t = 10\delta t$ using Esedoḡlu-Otto (EO) algorithm resulting in nucleation of orange cell at the blue triple junction; EO scheme with auction dynamics where orange cell splitting persists; and EO scheme with localized auction dynamics which preserves cell connectivity.

Supplementary Note 4. Numerical Tests

This note presents a series of numerical tests demonstrating the properties of the proposed scheme. In order to avoid jump-like behavior of errors inherent to algorithms using characteristic functions, we implement a simple idea for obtaining a sub-grid spatial accuracy in all numerical tests used to measure some type of error in numerical solution. Namely, after the diffusion step, we calculate the intersections of level sets with grid lines, use this information to obtain the ratio of area that each phase occupies in every grid cell, and based on these ratios construct a piecewise linear upgrade of the characteristic function that captures the interface more precisely. Moreover, to accelerate the computation, we optimized the auction dynamics step with bid priority queuing.

4.a. Numerical Convergence Test

We start with an investigation of the convergence order away from triple junctions. We consider the volume-preserving mean curvature flow of a three-phase initial configuration on a square domain $\Omega = [0,1] \times [0,1]$ with periodic boundary conditions, as shown in Supplementary Figure 4a, where both orange and blue phases consist of two disjoint circles of radii $R = 0.15$ and $r = 0.12$. We set $\sigma_{\mathrm{BO}} = \sigma_{\mathrm{BG}} = \sigma_{\mathrm{OG}} = 1.0$, which results in the larger circles growing as the smaller circles shrink and eventually vanish; thereby, satisfying the following system of differential equations:

$$\frac{dr}{dt} = -\frac{1}{r} + \frac{2}{r+R}, \qquad \frac{dR}{dt} = -\frac{1}{R} + \frac{2}{r+R} \tag{S.2}$$

For varying mesh-time configurations, we run our algorithm and compare its output with the precise approximation of the exact solution to the coupled differential equation (S.2) obtained by Runge-Kutta (RK4) method of order 4. We take the relative error of the radius of the resulting circles at around three-fourths of the extinction time of the smaller circles, that is, in our setup, at time $t = 0.01875$, which is the final time of the simulation. Supplementary Table 1 shows that the order of convergence of our algorithm is linear (cf., Jacobs et al.[20]). Supplementary Figure 4b,c shows log-log plot of the absolute error of radii of the resulting circles at $t = 0.01875$. One observes that taking too small time step relative to spatial grid size leads to an increase in error due to unwanted stagnation of interfaces.

Supplementary Figure 4: Basic convergence test for level set-based algorithm

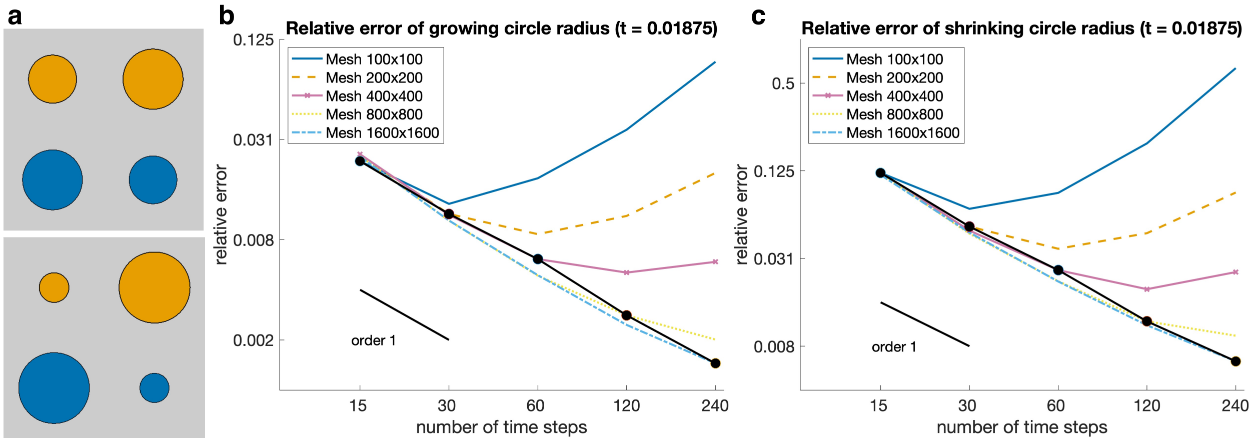

a, A three-phase initial configuration and its volume-preserving mean curvature evolution at time $t = 35\delta t$ with $\delta t = 0.0008$. **b,c**, Log-log plot of relative error of mean radius of larger circles (**b**) and smaller circles (**c**) at $t = 0.01875$ for varying configurations with $15, 30, 60, 120$ and 240 time steps and mesh resolutions: 100×100 (solid blue line), 200×200 (orange dashed line), 400×400 (purple solid line with cross), 800×800 (yellow dotted line), and 1600×1600 (sky blue dash-dotted line). Black points denote error values corresponding to space-time discretizations $(\delta x, \delta t)$ satisfying $\delta x = 8\delta t$ and demonstrate that the convergence order is linear. Raw data are provided in Supplementary Data 5.

Supplementary Table 1: Relative errors of level set-based algorithm for varying mesh-time configurations

mesh resolution	number of time steps	growing circle		shrinking circle	
		relative error	order	relative error	order
100×100	15	0.0231424	–	0.1208982	–
200×200	30	0.0111530	1.0531	0.0517636	1.2238
400×400	60	0.0059719	0.9012	0.0259983	0.9935
800×800	120	0.0027456	1.1211	0.0115676	1.1683
1600×1600	240	0.0014114	0.9600	0.0061546	0.9104

4.b. *Stationary Anisotropic Double Bubble Test*

To analyze the long-time behavior of the scheme, we consider a three-phase initial configuration on a square domain $\Omega = [0,1] \times [0,1]$ with periodic boundary conditions, as shown in Supplementary Figure 5a where two phases are identical squares sharing one common side of length 0.35. We denote $\mathcal{C}_1$ as the left square, $\mathcal{C}_2$ as the right square, and $\mathcal{C}_0$ as the remaining phase region, and set $\sigma_{01} = \frac{1}{2}\sqrt{3}$, $\sigma_{02} = 1$, and $\sigma_{12} = \frac{1}{2}$. Under volume-preserving anisotropic mean curvature flow, the stationary solution of such configuration is an anisotropic double bubble, which consists of three circular arcs meetings at two junction points with corresponding contact angles $\theta_0 = \frac{5\pi}{6}$, $\theta_1 = \frac{\pi}{2}$, and $\theta_2 = \frac{2\pi}{3}$.

Supplementary Figure 5: Numerical results of stationary double bubble test

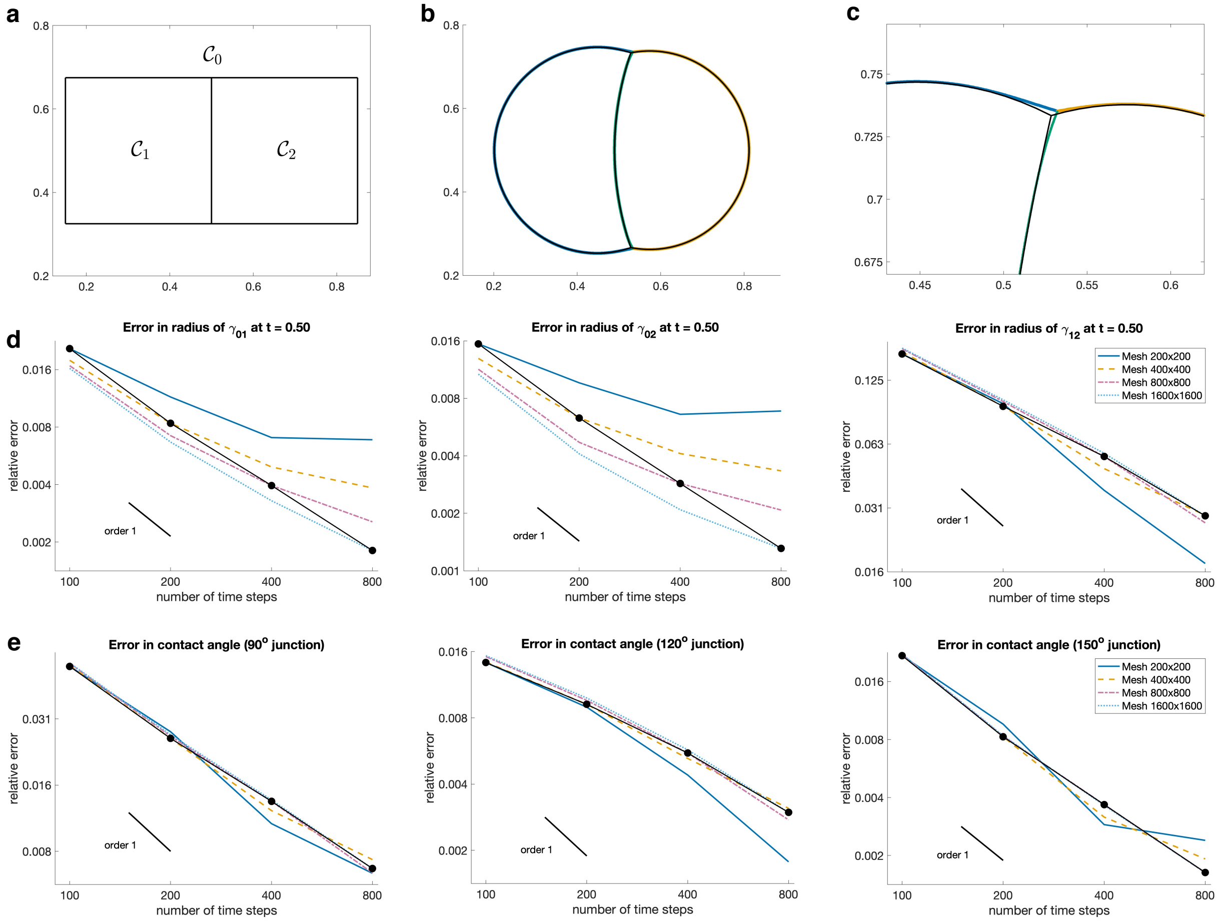

a, Three-phase initial configuration; **b**, its stationary numerical solution (color) using our algorithm with 1600×1600 mesh at $t = 0.5$ overlapping with the exact solution (black); and **c**, detail of its upper triple junction. **d,e**, Log-log plot of relative error in radius (**d**) of the best fitted circle to interface γ_{01}, γ_{02}, and γ_{12}; and relative error in contact angles (**e**) at the upper triple junction on phase $\mathcal{C}_1$, $\mathcal{C}_2$, and $\mathcal{C}_0$ for configurations with 100, 200, 400 and 800 time steps and mesh resolutions: 200×200 (blue solid line), 400×400 (orange dashed line), 800×800 (purple dash-dotted line), and 1600×1600 (sky blue dotted line). Black points denote error values obtained at space-time discretizations satisfying $\delta x = \delta t$, demonstrating convergence. Raw data are provided in Supplementary Data 6.

Using our algorithm, we take numerical stationary solution at time $t = 0.5$ for varying mesh-time configurations and compare these with the exact stationary solution. Using a least-squares fitting method to fit the resulting interface points to a circle, we compute the relative error of the radius of the best fitted circle on each interface. Supplementary Figure 5d shows the log-log plot of the relative error for each arc and mesh-time configuration, respectively. In addition, we check whether our numerical stationary solution satisfies the necessary angle conditions at the triple junction. We determine the numerical contact angles

using the normal vector to the best fitted circles at each triple junction. The obtained errors are displayed in Supplementary Figure 5e.

4.c. Contact Angle Analysis across Topological Change

Here we look into the ability of the algorithm to realize correct contact angles at triple junctions undergoing topology change. We consider a 4-cell aggregate of two types: 2 orange and 2 blue cells on a square domain $\Omega = [0,1] \times [0,1]$ with periodic boundary conditions (see Supplementary Figure 6a). Taking $\sigma_{\mathrm{BB}} = \sigma_{\mathrm{OO}} = \sqrt{2}$ and $\sigma_{\mathrm{BO}} = 1$ evolves the configuration in such a way that at both middle junctions, the contact angle opening towards a blue cell changes from the initial angle 180° to 90°, until a topological change occurs, that is, the blue cells intercalate and the orange cells split. At this point, the contact angle changes from 90° to 135° and the cell-cell junctions deform until they reach a stationary state.

Supplementary Figure 6: Numerical test on topology change.

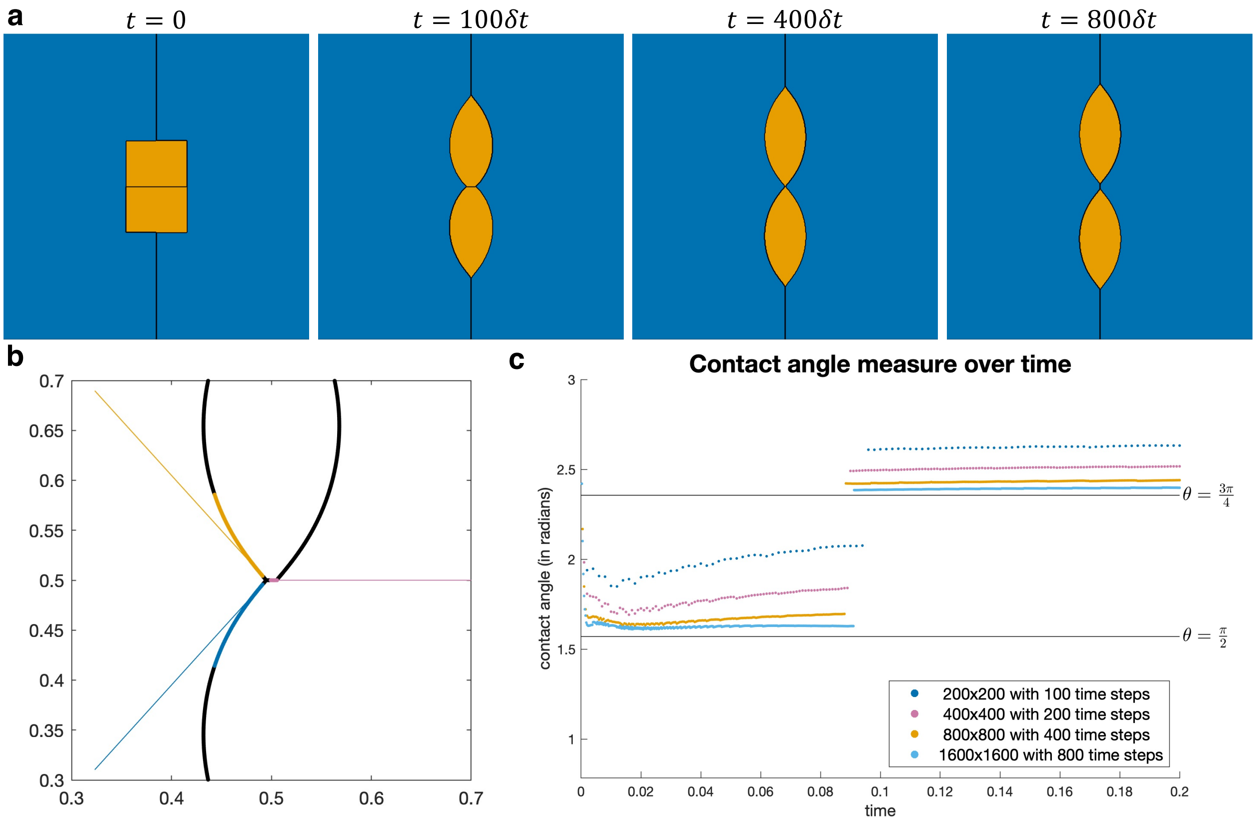

a, An initial configuration of two cell types: 2 orange and 2 blue cells, and snapshots its evolution using the level set-based approach with $\delta t = 2.5 \times 10^{-4}$ with interfacial tensions $\sigma_{\mathrm{BB}} = \sigma_{\mathrm{OO}} = \sqrt{2}$ and $\sigma_{\mathrm{BO}} = 1$ on a 500×500 mesh. **b**, The portion of the interface at time $t = 0.05$, whose distance away from the junction falls on the interval $(0.005, 0.10)$, used to calculate the contact angles for the 800×800 mesh. **c**, Evolution of the magnitude of contact angle measured across topology change, shown for mesh-time configurations: 200×200 with 100 time steps (blue), 400×400 with 200 time steps (purple), 800×800 with 300 time steps (orange), and 1600×1600 with 400 time steps (sky blue). Raw data are provided in Supplementary Data 7. Theoretically, the value should jump from $\pi/2$ to $3\pi/4$ radians, as shown by the black lines.

We run our level set-based algorithm until time $t = 0.2$ for varying mesh-time configurations (see Supplementary Table 2). To measure the numerical contact angles, we find the best fitted circle to each interface (excluding the junction point) and compute the contact angles using their tangent lines. In particular, we take the portion of the interface whose distance away from the junction falls on the interval

(0.005, 0.10), as shown in Supplementary Figure 6b. We then compute the L^1 error on contact angles in the time interval from $t = 0.05$ to $t = 0.15$, and display the results in Supplementary Table 2 and Supplementary Figure 6. We see that error decreases as both mesh and time are further refined. Supplementary Figure 6 also shows that the numerical time when topological change occurs differs slightly for each mesh-time configuration.

Supplementary Table 2: L^1-error of contact angle measure for varying mesh-time configurations

mesh resolution	number of time steps	contact angle	
		L^1 error	order
200×200	100	0.0289831	-
400×400	200	0.0186973	0.6324
800×800	400	0.0088110	1.0855
1600×1600	800	0.0044431	0.9877

4.d. Evolving Double Bubble Test

Using the same initial configuration as in Supplementary Figure 5 where two phases are identical squares sharing one common side of length 0.35, we study how well our algorithm captures the dynamics of the evolution. Since analytical solution is not available, we implemented a front-tracking algorithm with automatic redistribution of points along interfaces that can deal also with triple junctions, and used its high-resolved solution at time $t = 0.024$ as an accurate approximation of the true solution. At this time, the double bubble is powerfully evolving and far from the stationary state.

Supplementary Table 3: Error of evolving double bubble vs. front-tracking scheme

mesh resolution	time step size	error	order
100×100	0.0080	0.006570	-
200×200	0.0040	0.004938	0.4118
400×400	0.0020	0.003843	0.3620
800×800	0.0010	0.003190	0.2685
1600×1600	0.0005	0.002782	0.1975

Supplementary Figure 7: Numerical test on multiphase dynamics.

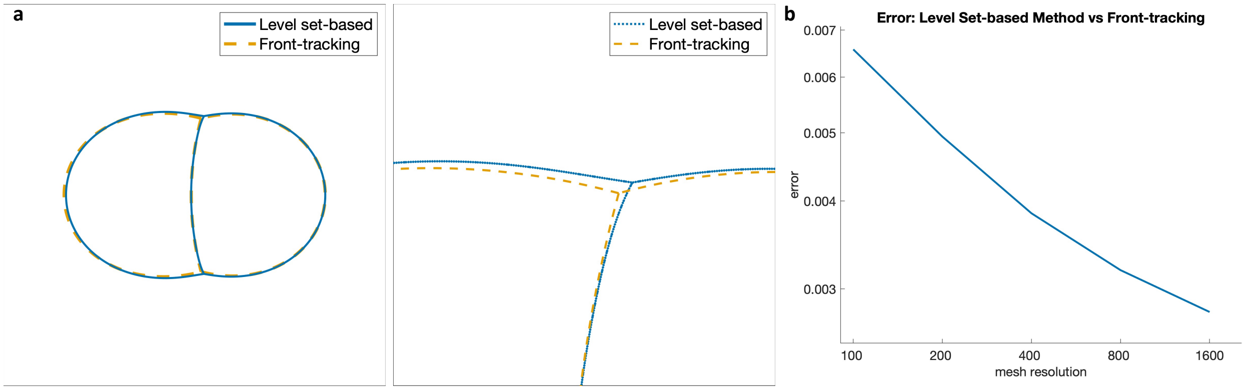

a, Snapshot of an evolving anisotropic double bubble at time $t = 0.024$ using level set-based method (blue solid line) and front-tracking method (orange dashed line) and detail of their upper triple junctions. **b**, Log-log plot of the error of evolving double bubble vs. front-tracking method for varying mesh-time configurations provided in Supplementary Table 3.

Supplementary Table 3 and Supplementary Figure 7 show that the error, defined as the area of the symmetric set difference of the right bubble's front-tracking approximation and its realization by our

algorithm, decreases with mesh-time grid refinement. However, due to the presence of triple points the overall convergence rate falls below linear.

Supplementary Note 5. Comparison with Vertex Dynamics

To highlight the ability of the algorithm to deal with topology changes, such as cell intercalations, let us delve into cellular pattern formations in developmental stages in the olfactory epithelium (OE). Katsunuma et al.[16] hypothesized that heterophilic trans-interaction between nectin-2 on olfactory cells (OCs) and nectin-3 on supporting cells (SCs) promote recruitment in the cell-cell junction of the cadherin-catenin complex whose representative marker, β-catenin indicates the differential adhesions required to drive self-organized cell movements in OE. Following the differential adhesion hypothesis[11,12], Katsunuma el al.[16] simulated two cases of cellular patterns: one with adhesion strengths $\alpha_{\mathrm{SS}} = \alpha_{\mathrm{SO}} = 1.0$, and $\alpha_{\mathrm{OO}} = 0.533$; while the other had a weaker adhesion $\alpha_{\mathrm{SO}} = 0.833$. Using the vertex dynamics model with $\sigma_{ij} = \alpha_{ij}^{-1}$, they were able to confirm that the first case leads to cellular intercalation, while the second does not; thereby, supporting their idea that differential adhesion in heterotypic cell-cell junctions drives cell intercalations.

In order to compare the performance of the standard vertex dynamics algorithm and our scheme, we consider an initial cellular aggregate of 62 SCs (blue) and 2 OCs (orange) cells of almost equal volumes, similar to that in[16], with periodic boundary conditions on the square domain $\Omega = [0, 1] \times [0, 1]$ (see Supplementary Figure 8). We simulate both cases using the vertex dynamics model and our proposed scheme, with the same time step size $\delta t = 0.0003$. For the vertex dynamics model, we employ the same potential as in Katsunuma et al.[16] with two types of cell volume penalty ($\rho = 1000$ and $\rho = 500$) and minimum threshold distances $\tau = 10^{-3}$ for T1-transition. Moreover, for our level set-based approach, we discretize the domain uniformly into $M = 1000 \times 1000$ points. Numerical results are shown in Supplementary Figure 8 – note here that the times where the snapshots are taken at largely different for each method (see also Supplementary Movies 8, 9, and 10).

We observe that both methods do lead to cellular intercalation when adhesion strengths $\alpha_{\mathrm{SS}} = \alpha_{\mathrm{SO}} = 1.0$, and $\alpha_{\mathrm{OO}} = 0.533$ are set. However, essential differences are found in the results. First, the time needed for the intercalation to occur in the vertex dynamics algorithm heavily depends on the minimum T1-transition threshold distance τ, which can be seen by following the orange line in Supplementary Figure 8a – the edge length starts to increase when this line hits the level τ. In other words, a small change in τ may result in largely different times of intercalation, which in turn may have serious impact on the global dynamics of the aggregate. Second, and probably the most prominent difference between the two methods lies in the time rate of change of the junction length, i.e., the vertex dynamics motion is reluctant to undergo topology changes. Lastly, inspecting Supplementary Figure 8a, it is clear that both methods yield different final shapes of the intercalated cells. We remark that the jump-like behavior of the junction length in the level-set based method (black line), which can be observed in Supplementary Figures 8 and 9, is due to fact that the level set method is performed on a fixed grid causing sudden changes of the length as a tricellular point jumps from one grid cell to another. On the other hand, the meshless vertex dynamics moves the tricellular points freely in space and hence yields smoothly changing lengths. However, the jumps observed in the level-set based method diminish with the refinement of the grid.

To confirm whether this aspect of vertex dynamics is pertinent to topology changes such as intercalation, we have compared the evolution of junction lengths with cases where no intercalation occurs. Results for one such setting are shown in Supplementary Figure 8b, where the difference in adhesion strengths (having $\alpha_{\mathrm{SS}} = 1.0, \alpha_{\mathrm{SO}} = 0.833, \alpha_{\mathrm{OO}} = 0.533$) drives the OCs towards intercalation but is not strong enough for the intercalation to occur. Another test case, depicted in Supplementary Figure 8c sets all adhesion strengths equal to 1.0 and considers an initial configuration with unnaturally long junction between the red cells. One observes that the dynamics in this last case is similar for both methods leading to approximately the same stationary junction length, although the vertex dynamics model evolves slightly slower. In the “almost intercalating” test case of Supplementary Figure 8c, the vertex dynamics model lags behind the level set-based scheme and converges to a largely different stationary length of the junction, which also strongly

depends on the parameters of the vertex dynamics model, in this case, the volume penalty. Meanwhile, in all test cases, the level set-based algorithm swiftly approaches the steady state configuration, irregardless of the presence of intercalation, which conforms with its convergence proof[21]. From these findings, we may conclude that vertex dynamics model tends to slow down the evolution when topology changes are to take place, contrary to what is expected from the energy minimization principle.

Supplementary Figure 8: Vertex dynamics vs. Level set-based model.

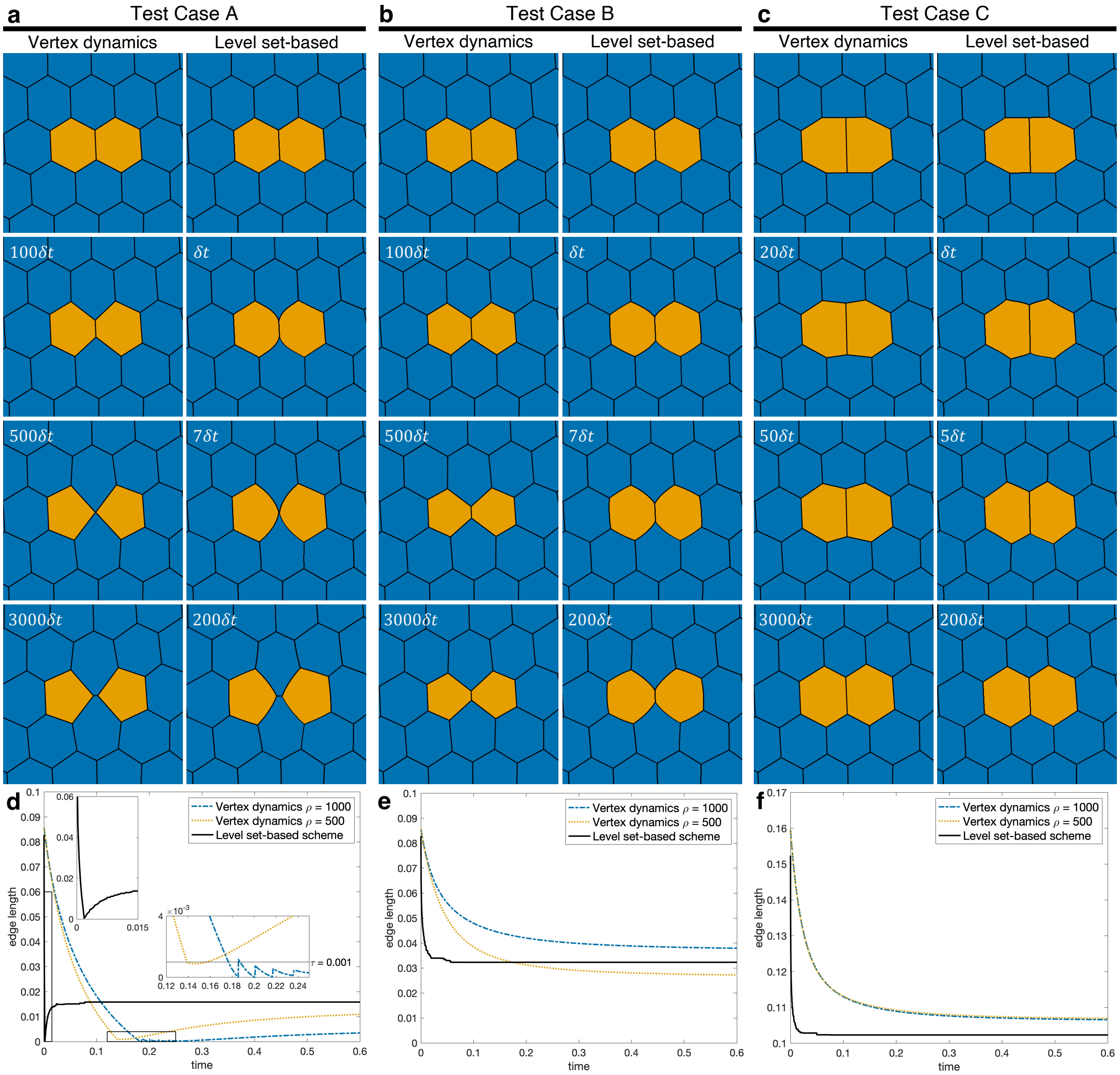

a-c, Zoomed-in configuration of an initial aggregate of 62 SCs (blue) and 2 OCs (orange) and snapshots of its evolution via vertex dynamics model with $\rho = 500$ (left column) and level set-based approach (right column) for three test cases of adhesion strengths: (**a**) test case A with $\alpha_{SS} = \alpha_{SO} = 1.0$ and $\alpha_{OO} = 0.533$ leading to cellular intercalation; (**b**) test case B with $\alpha_{SS} = 1.0$, $\alpha_{SO} = 0.833$ and $\alpha_{OO} = 0.533$ where no intercalation occurs; and (**c**) test case C with $\alpha_{SS} = \alpha_{SO} = \alpha_{OO} = 1.0$ which shortens OO-junction. **d-f**, Plot showing the shrinkage of the OO-junction length (and the formation of a new SS-junction for the case where cellular intercalation occurs), generated using vertex dynamics model expressing different volume penalties: $\rho = 1000$ (blue dash-dotted line) and $\rho = 500$ (orange dotted line); and our level set-based approach (black solid lines) for test cases A, B, and C, respectively. Raw data are provided in Supplementary Data 8.

This difference between the models is mainly caused by three factors, as follows. The first one is due to the different form of their energies, since the vertex model includes not only the surface energy but also other terms such as volume penalty term, etc. Secondly, the correct cell contact angles are not realized in the vertex dynamics model, particularly for cell-cell junctions that should be theoretically curved, which may alter succeeding cell dynamics. This, on the contrary, does not occur in the level set-based approach, as it is theoretically proved to satisfy the tricellular angle condition. Lastly, the vertex dynamics model can only take a restricted set of paths to minimize its energy. Indeed, since the level set-based method allows for arbitrary shapes of junctions, contrary to the vertex model where only polygonal shapes are allowed, it is able to follow the gradient descent of the energy correctly; while the vertex dynamics model is delayed (and for some cases, completely stopped) by having to take only polygonal deformations.

We elaborate on the first difference mentioned in the previous paragraph. In our model, the energy consists only of surface energy, and the volume preservation is realized through the constraint condition. Thus, the cell sizes are preserved precisely. On the other hand, in the vertex model, the energy is defined as the sum of surface energy and volume penalty term, while it is necessary to use a large value of penalty coefficient ρ in order to preserve the cell sizes to some extent. The effect of the surface energy term is then relatively weakened when ρ is large. In this way, the level set-based approach allows us to focus on the effect of surface or adhesion energy, unlike the vertex model where it is difficult to separate and correctly understand this effect (see Supplementary Figure 9 for a detailed analysis). This provides a typical example of the importance of reducing model parameters: the vertex model does not facilitate the understanding of the adhesion mechanism because the influence of its two additional parameters, namely the volume penalty coefficient ρ and threshold distance for T1 transition τ, cannot be easily analyzed. Indeed, as one can see in Supplementary Figure 8a, mere doubling of the volume penalty coefficient ρ leads to a completely different behavior of the edge length. The oscillations of the edge length for the stronger volume penalty represent a tug of war triggered by the T1 transition between the elastic volume term and the surface adhesion term in the energy.

Supplementary Figure 9: Evolution of energy and cell volumes for the simulation in test case A.

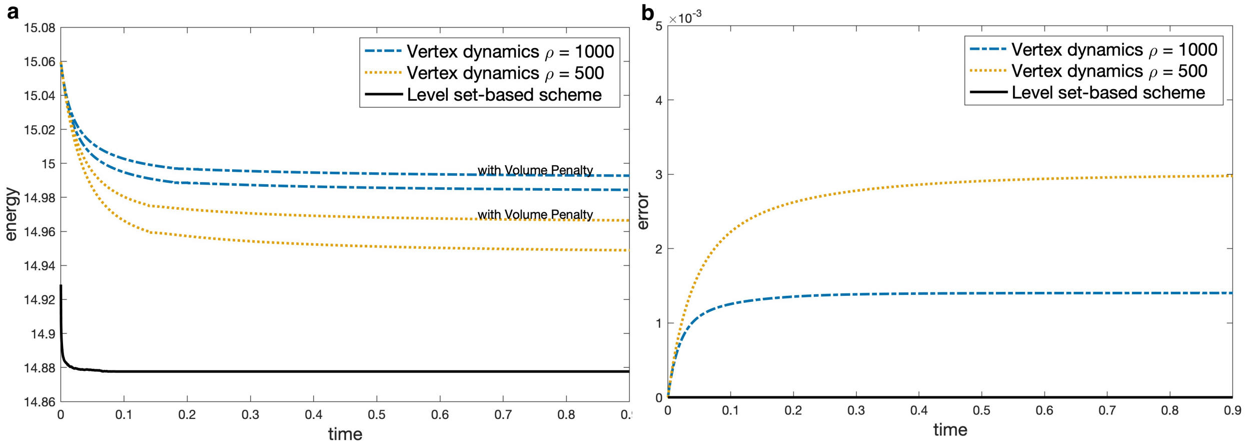

a, The total energy (upper blue dash-dotted line) and its surface energy component (lower blue dash-dotted line) of the vertex model for volume penalty $\rho = 1000$ and analogous results for penalty $\rho = 500$ (orange dotted line). The black solid line shows the evolution of energy of level set-based method. The discrepancy in initial energy for both methods is caused by the different representation of cell shapes. **b**, The evolution of error in cell volumes defined at time t_k as the maximum absolute deviation of cell volumes from the prescribed volume, where the volume is calculated as polygonal area in the vertex dynamics model ($\rho = 1000$: blue dash-dotted line, $\rho = 500$: orange dotted line), and by $v_i^k(\delta x)^2$ in the level set based model (solid black line). Raw data are provided in Supplementary Data 8.

Supplementary Note 6. Computational Cost of the Algorithm

Here we perform several numerical experiments to assess the computational cost of the level set-based algorithm and confront it with a different representative method, namely the vertex dynamics. Contrary to the vertex dynamics, where the x, y-coordinates of the vertices are the only degrees of freedom, the level set method requires determining functional values at every point of the two- or three-dimensional grid. Therefore, the level set method solves a higher-dimensional discrete problem, and in this sense is expected to have theoretically higher computational cost, especially on fine grids. Meanwhile, as the partial differential equation to be solved is a simple heat equation, the application of FFT allows its fast solution, as detailed in the Methods section. Moreover, the intercalation example in test case A (Supplementary Figure 8a) shows that the correct realization of gradient flow by the level set methods requires substantially smaller number of time steps to reach the equilibrium compared to the stiff evolution by vertex dynamics. Hence, the theoretically higher computational cost does not always imply longer computational time needed to reach the required solution.

Supplementary Table 4: CPU time (in seconds) of level set-based simulation of volume-preserving mean curvature flow

mesh resolution	number of time steps					
	15	30	60	120	240	480
100×100	0.33	0.41	0.58	0.91	1.59	2.93
200×200	0.83	1.15	1.80	3.10	5.59	10.91
400×400	2.34	3.86	6.83	12.46	23.54	45.14
800×800	7.36	12.79	24.13	47.09	88.72	168.19
1600×1600	28.21	51.79	99.84	195.49	388.63	768.01

Supplementary Figure 10: Computational cost of level set-based algorithm.

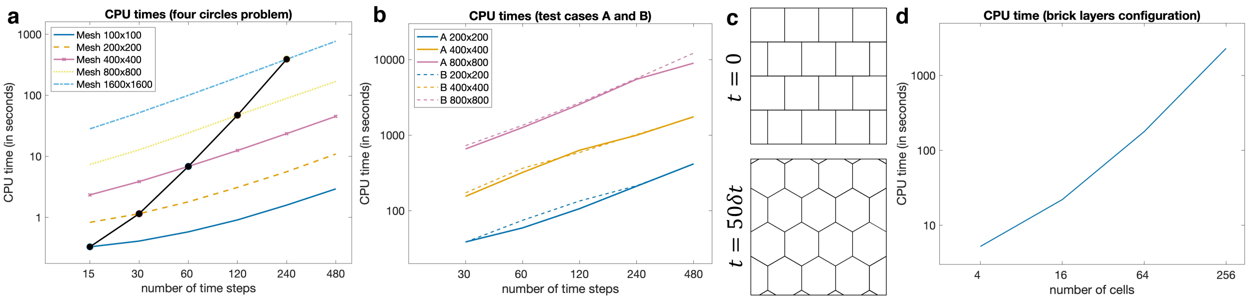

a, Log-log plot of CPU time (in seconds) of level set-based simulation of volume-preserving mean curvature flow provided in Supplementary Table 4 for configurations with $15, 30, 60, 120, 240$, and 480 time steps and mesh resolutions: 100×100 (solid blue line), 200×200 (orange dashed line), 400×400 (purple solid line with cross), 800×800 (yellow dotted line), and 1600×1600 (sky blue dash-dotted line). **b**, Log-log plot of CPU time (in seconds) of level set-based simulation of test cases A (solid line) and B (dashed line) provided in Supplementary Tables 5 and 6, respectively for configurations with $30, 60, 120, 240$, and 240 time steps and mesh resolutions: 200×200 (blue), 400×400 (orange), and 800×800 (purple). Black points show the tendency of CPU times when number of discrete points is doubled both in time and the two spatial directions. The slope of black lines is approximately 8, suggesting linear dependence. **c**, Initial configuration of 16 brick-like cells with periodic boundary condition and snapshot of its evolution after 50 time steps using 800×800 mesh and time step $\delta t = 0.001$. **d** Log-log plot of the dependence of CPU time on the number of cells provided in Supplementary Table 7.

Since it is not possible to cover all such case-dependent aspects, we use simple examples to report on the generic computational cost of both algorithms. All CPU times and memory usage tests reported in this section were conducted on a MacBook with 2 GHz quad-core Intel Core i5 processor. Using the same setup as in Supplementary Note 4.a, we measure runtime of our level set-based algorithm in terms of CPU time in seconds for varying mesh-time configurations. Supplementary Table 4 and Supplementary Figure 10a show

a moderate computational cost for finer mesh-time configurations over the vertex dynamics scheme, which is computationally cheaper in the sense that computations are only done at the vertices, contrary to all mesh nodes for the level set-based method. On the other hand, the cost increases linearly in both methods with respect to refinements: in time and each spatial direction for level set-based method, and in time and in number of vertices in vertex dynamics. The last statement is supported by Supplementary Figure 11, which shows the dependence of CPU time on time step size in vertex dynamics applied to test cases A and B from Supplementary Note 5. Here CPU time was measured until a stopping criterion based on a stationary state is met; namely, distance by which each vertex in the network moved was measured in every time step and the code was terminated when all vertices moved by less than 10^{-12}. The CPU times are longer for test case A, where topology change has to be detected and implemented by a T1 swap. The cost difference from test case B, which does not involve any topology change, is not large but it is expected that it will become prominent when many T1 swaps are required during the evolution.

Supplementary Figure 11: Computational cost of vertex dynamics algorithm.

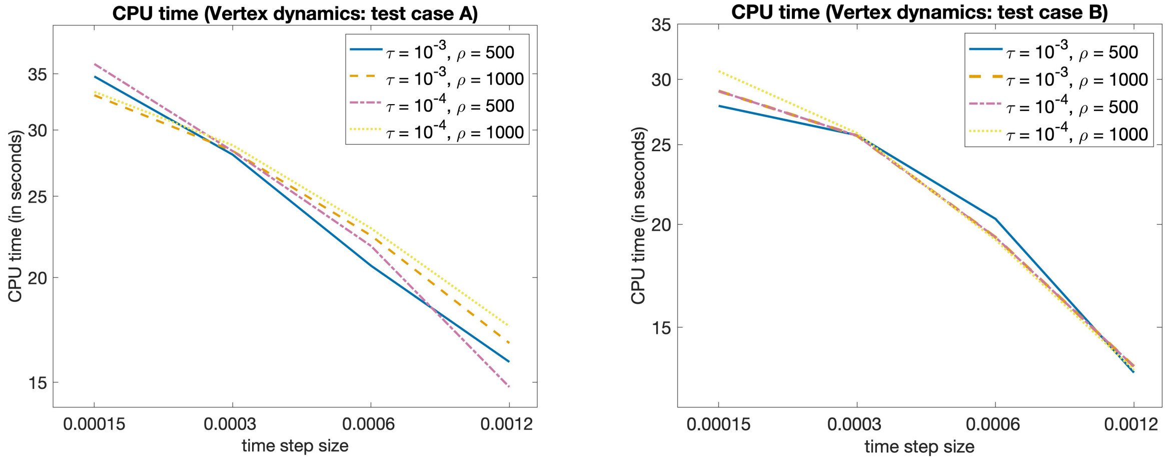

Log-log plots of CPU times in executing the vertex dynamics algorithm for four set of parameters: $\tau = 10^{-3}, \rho = 500$ (blue solid line); $\tau = 10^{-3}, \rho = 1000$ (orange dashed line); $\tau = 10^{-4}, \rho = 500$ (purple dash-dotted line); and $\tau = 10^{-4}, \rho = 1000$ (yellow dotted line) on test case A (with topology change) and B (without topology change), showing increased cost when T1-swap is present. Raw data are provided in Supplementary Data 9.

Supplementary Table 5: CPU time (in seconds) of level set-based algorithm for test case A

mesh resolution	number of time steps				
	30	60	120	240	480
200×200	38.60	59.26	106.51	211.13	416.63
400×400	154.99	321.23	631.96	997.70	1751.74
800×800	656.70	1262.54	2555.67	5491.67	8978.75

Supplementary Table 6: CPU time (in seconds) of level set-based algorithm for test case B

mesh resolution	number of time steps				
	30	60	120	240	480
200×200	38.42	74.73	134.55	214.20	414.06
400×400	172.37	364.85	590.31	1018.51	1733.04
800×800	726.08	1351.05	2685.12	5641.49	12183.31

In order to check whether level set-based simulation of configurations with topological changes affect computational cost, we employ test cases A and B from Supplementary Note 5 and measure runtime of our algorithm in terms of CPU time in seconds for varying mesh-time configurations. Supplementary Tables 5

and 6 as well as Supplementary Figure 10b indicate that there is no essential difference in computational cost between simulations with and without topological changes.

In addition, we investigated the dependence of computational cost on the number of simulated cells. The domain $\Omega = [0,1] \times [0,1]$ was discretized using 800×800 grid points, time step was set to $\delta t = 0.001$ and initial configuration was formed by "layers of bricks" with periodic boundary condition as shown in Supplementary Figure 10c. This initial network was constructed for 4, 16, 64 and 256 cells and evolved under uniform and constant interfacial tension and periodic boundary conditions for 50 time steps, when it approximately reached the stable honeycomb pattern shown in Supplementary Figure 10c. Supplementary Figure 10d and Supplementary Table 7 show the required CPU times and memory for each of the four simulations. A quadratic function fitted tightly to the CPU time curve showing that the CPU times are proportional to the square of the number of cells. This matches with the theoretical prediction of quadratic complexity of auction dynamics[20], and implies higher demands on computational resources than the linear dependence of vertex dynamics. Analogous behavior was obtained for different choices of discretization parameters.

Supplementary Table 7: Dependence of computational cost of level set-based algorithm on number of cell regions

number of cells	CPU time (s)	memory (kB)
4	5.204	25,784
16	22.007	85,812
64	177.926	326,384
256	2300.612	970,388

Lastly, we measure memory usage in kilobytes of our algorithm for varying mesh-time configurations using test cases A and B from Supplementary Note 5. Supplementary Table 8 shows that there is no difference in memory usage between test cases A and B. On the other hand, vertex dynamics solely requires storing of small arrays of the size proportional to the number of vertices. In our test cases A, B we have 64 cells with 128 vertices, entailing memory usage as small as approximately 45 kB, independently of choice of parameters. Our level set-based algorithm has a substantially higher memory usage – the price for expressing complex cell geometries.

Supplementary Table 8: Memory usage (in kilobytes) of level set-based algorithm for various mesh configurations

mesh resolution	test case A	test case B
200×200	84,954	84,311
400×400	326,984	327,640
800×800	1,300,704	1,299,728

Supplementary Note 7. Cellular Mosaic Patterns

Among the phenomena that occur in a heterotypic aggregate of embryonic cells are cell sorting, mixing, and formation of checkerboard patterns. In epithelial tissues, for example, when cells expressing different types of cadherin – a homophilic cell adhesion molecule – are mixed, these cells form separate aggregates[22]. Similar segregation of cells occurs if cells expressing different amounts of the same cadherin are mixed[23]. From these observations, cadherin quantity and affinity are thought to control tissue segregation and assembly. In contrast to cadherins, nectins prefer heterotypic binding to homotypic one, and their heterophilic interactions produce stronger cell-cell adhesions than their homophilic interactions. Owing to these properties of nectins, cells in mixed cultures expressing different nectins became arranged in a mosaic pattern[24] (see Supplementary Figure 12).

Cell sorting begins with the formation of smooth chains of cells, followed by shortening of these chains into round masses, and finally annealing of the resulting masses[25]. From the viewpoint of the differential

interfacial tension hypothesis[13], self-rearrangement of embryonic cells and tissues are said to be driven by differences in interfacial tensions. Finite element-based simulations[7,25] showed that for an aggregate of two cell types, say, blue and orange cells, sorting occurs only when interfacial tensions satisfy $\sigma_{\mathrm{BO}} > \frac{1}{2}(\sigma_{\mathrm{BB}} + \sigma_{\mathrm{OO}})$; meanwhile a sufficient condition for mixing is $\sigma_{\mathrm{BO}} < \frac{1}{2}\sigma_{\mathrm{BB}}$ or $\sigma_{\mathrm{BO}} < \frac{1}{2}\sigma_{\mathrm{OO}}$.

Supplementary Figure 12: Two different cell sorting mechanisms.

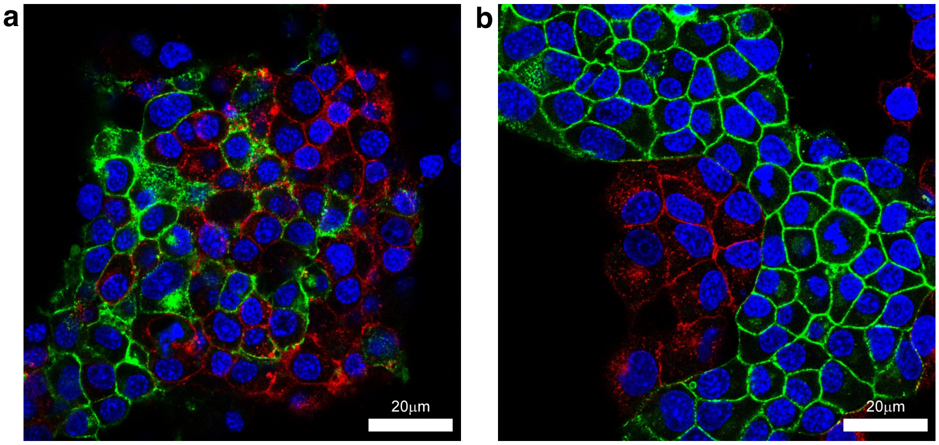

a, Mosaic pattern of a mixed culture of cells expressing different nectins (green: nectin-1 (AF297665.1); red: nectin-3 (NM_ 021495.4); blue: cell nucleus). **b**, Segregated pattern of a mixed culture of cells expressing different cadherins (green: N-cadherin (NM_007664.5); red: E-cadherin (NM_009864.3); blue: cell nucleus).
Stable transfectants of Neuro2a cells expressing mouse nectin-1 or -3, mouse E-cadherin or N-cadherin were generated by standard transfection methods. Cultures were maintained in DMEM/F-12 with 10% fetal calf serum. In the co-culture experiments, Neuro2a transfectants were dissociated into single cells, mixed at a ratio of 1:1, and cultured for 36 hrs.

Supplementary Figure 13: Cell sorting via level set-based model.

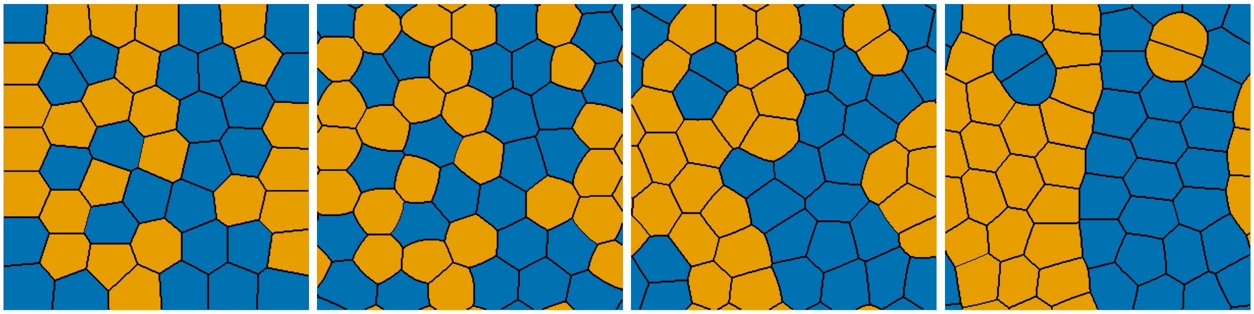

Initial aggregate of 25 blue and 25 orange cells with interfacial tensions $\sigma_{\mathrm{BB}} = 0.6$ and $\sigma_{\mathrm{OO}} = 1.0$; its evolution generated by the level set-based approach for partial mixing when $\sigma_{\mathrm{BO}} = 0.7$; partial sorting when $\sigma_{\mathrm{BO}} = 1.1$; and strong sorting when $\sigma_{\mathrm{BO}} = 2.0$.

We recreate these simulations using our level set-based approach. Consider an initial aggregate of 50 similar-sized cells where cell types are randomly assigned to 25 blue and 25 orange cells (see Supplementary Figure 13 and Supplementary Movie 11). We discretize the domain $\Omega = [0,1] \times [0,1]$ uniformly into $M = 300 \times 300$ points, consider periodic boundary conditions, and set time step $\delta t = 0.0008$. In this setup, we take interfacial tensions $\sigma_{\mathrm{BB}} = 0.6$ and $\sigma_{\mathrm{OO}} = 1.0$ over 300 time steps. We observe that when $\sigma_{\mathrm{BO}} = 0.7$, partial mixing of blue and red cells occurs. Moreover, increasing this interfacial tension to $\sigma_{\mathrm{BO}} = 1.1$ leads to partial sorting of different cell types; and further increasing to $\sigma_{\mathrm{BO}} = 2.0$ results in strong sorting. Here we scale by 5% the interfacial tensions considered by Brodland[13]. Hence, our method is able to produce similar results as those of the finite element-based simulations in Figure 5 of Brodland[13]. The important difference is that our

level set-based method naturally handles topological changes; while the FEM-based approach requires an ad hoc scheme, in particular, a boundary "flip" algorithm to handle cellular intercalations, which may cause problems for certain cellular configurations, leading to unnaturally distorted shapes of cells, in the same way as in the vertex dynamics model.

On the other hand, employing the viewpoint of differential adhesion hypothesis, Katsunuma et al.[16] hypothesized that relative intensities of β-catenin accumulations in mixed cultures of cells expressing nectin-2 and N-cadherin (blue cells in Supplementary Figure 14) with various transfectants (orange cells in Supplementary Figure 14) lead to different mosaic cellular patterning. In particular, a segregated pattern is formed when mixed with cells expressing nectin-2, N-cadherin, and E-cadherin where the adhesion strengths satisfy $\alpha_{OO} > \alpha_{BO} > \alpha_{BB}$; a checkerboard pattern when mixed with cells expressing nectin-3 and N-cadherin resulting in $\alpha_{BO} > \alpha_{RR} = \alpha_{BB}$; and a football (kagome) pattern when mixed with cells expressing nectin-3, N-cadherin, and E-cadherin where $\alpha_{BO} = \alpha_{OO} > \alpha_{BB}$. To confirm these hypothetical profiles of synergistic actions of nectins and cadherins on cellular patterning, we use our level set-based scheme and simulate the corresponding cell motions. In our simulations involving adhesion as the physical parameter, we always set the coefficients σ in the energy formula (equation (1) in the main manuscript) as the reciprocal of the cell-cell adhesion strength α, i.e., $\sigma = \alpha^{-1}$.

Supplementary Figure 14: Cellular patterns via level set-based model according to hypothetical profiles of synergistic actions of nectins and cadherins.

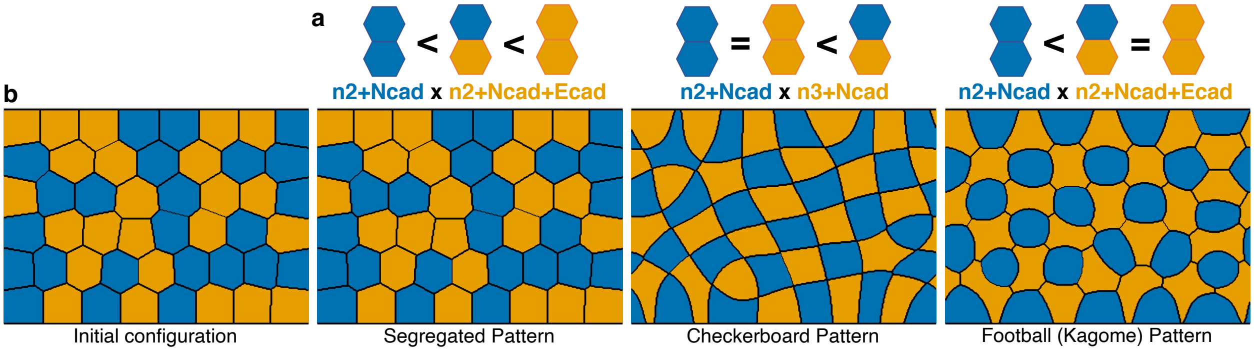

a, Schematic diagrams of the relative intensities of the β-catenin accumulations (corresponding to relative adhesion strengths) in mixed cultures of various transfectants, resulting in different mosaic patterns (adapted from Journal of Cell Biology, ©2016 Katsunuma et al.[16]). **b**, Initial 48-cell aggregate consisting of 24 blue cells expressing nectin-2 and N-cadherin; and its evolution via level set-based approach resulting in different mosaic patterns: a segregated pattern with orange cells expressing nectin-2, N-cadherin, and E-cadherin; a checkerboard pattern with orange cells expressing nectin-3 and N-cadherin; and football (kagome) pattern with orange cells expressing nectin-3, N-cadherin, and E-cadherin.

We consider an initial 48-cell aggregate where cell types are randomly assigned to 24 blue and 24 orange cells (see Supplementary Figure 14 and Supplementary Movie 12). We take a computational domain $\Omega = [0,1] \times [0,1]$ discretized uniformly into $M = 300 \times 300$ points and set time step $\delta t = 0.001$. On the left and right boundaries, we impose periodic boundary conditions; while on the top and bottom boundaries, we prescribe a fixed adhesion strength of 0.10. Moreover, we consider time-dependent cell-cell adhesion strengths, which change piecewise linearly in time starting from $\alpha_{BB} = \alpha_{BO} = \alpha_{OO} = 1.0$ over 1500 time steps. More precisely, we increase the adhesion strengths starting from 1.0 in increments of size 0.25 keeping them constant for 150 time steps each time before the next increase, until they reach the value 3.0, which is kept constant for 450 time steps in order to completely equilibrate. Observe that when adhesion strengths $\alpha_{BB} = \alpha_{BO} = 1.0$ are kept constant, while α_{OO} is linearly changed to 3.0, the blue and orange cells segregate creating a final configuration similar to that produced by cell sorting. In particular, since α_{OO} is large, the orange cells strongly adhere to each other and sort out the blue cells. Moreover, for target adhesion strengths $\alpha_{BB} = \alpha_{OO} = 1.0$ and $\alpha_{BO} = 3.0$, the stationary solution forms a checkerboard pattern. Note that a perfect checkerboard pattern is not attained due to the topological limitations near the boundary but orange and blue cells are still distributed in a fully alternating pattern. Finally, changing adhesion strengths

to $\alpha_{\mathrm{BB}} = 1.0$ and $\alpha_{\mathrm{BO}} = \alpha_{\mathrm{OO}} = 3.0$ results in a football pattern. However, since there are equal number of cells for each type, only four blue cells surround each orange cell and vice versa. If the initial aggregate has more orange cells, enough for six of them to surround one blue cell, the final pattern will be much closer to the standard football pattern.

Supplementary Note 8. Cell Engulfment and 3D Simulations

In this note, we look into embryo morphogenesis where mammalian embryo self-organizes into a blastocyst, consisting of epithelial layer encapsulating the inner-cell mass[26]. Since asymmetrically divided 8-cell-stage blastomeres encompass both the morphogenesis and fate specification of the whole embryo, it is enough to consider a cell doublet as an initial configuration, which results in the blue cell (B) enveloping its neighboring orange cell (O) in an entosis-like process within a white medium (W). Maitre et al.[26] showed that cells internalize only when differences in surface contractility exceed a predictable threshold, in particular, when $\sigma_{\mathrm{OW}} \geq \sigma_{\mathrm{BW}} + \sigma_{\mathrm{BO}}$, see equation (S16) in the Supplement of their paper.

We consider an initial configuration of a cell doublet on a cubical domain $\Omega = [0,1] \times [0,1] \times [0,1]$ discretized uniformly into $M = 300 \times 300 \times 300$ points. We present a three-dimensional numerical simulation using our level set-based scheme with time step $\delta t = 0.005$ and interfacial tensions satisfying the cell internalization condition. In particular, we start with $\sigma_{\mathrm{BO}} = 1.6$, and $\sigma_{\mathrm{BW}} = \sigma_{\mathrm{OW}} = 1.0$, then decrease the compaction parameter α from 0.8 to 0.25 through the reduction of σ_{BO} to 0.5, followed by an increase in tension asymmetry δ from 1.0 to 1.6 through the change of σ_{OW} to 1.6. The value of $\sigma_{BW} = 1.0$ is kept constant throughout the simulation. Results presented in Supplementary Figure 15 and Supplementary Movie 13 confirm that our method produces similar results as in Maitre et al.[26], and more importantly, it can handle simulations in higher dimensions without technical or theoretical complications. Indeed, the algorithm remains the same, it is only solved on a 3-dimensional spatial mesh. This naturally entails a heavy increase in the demand on computational resources and thus simulation of larger number of cells in 3D will require parallelization and other improvements in the basic code.

Supplementary Figure 15: Cell internalization via level set-based model.

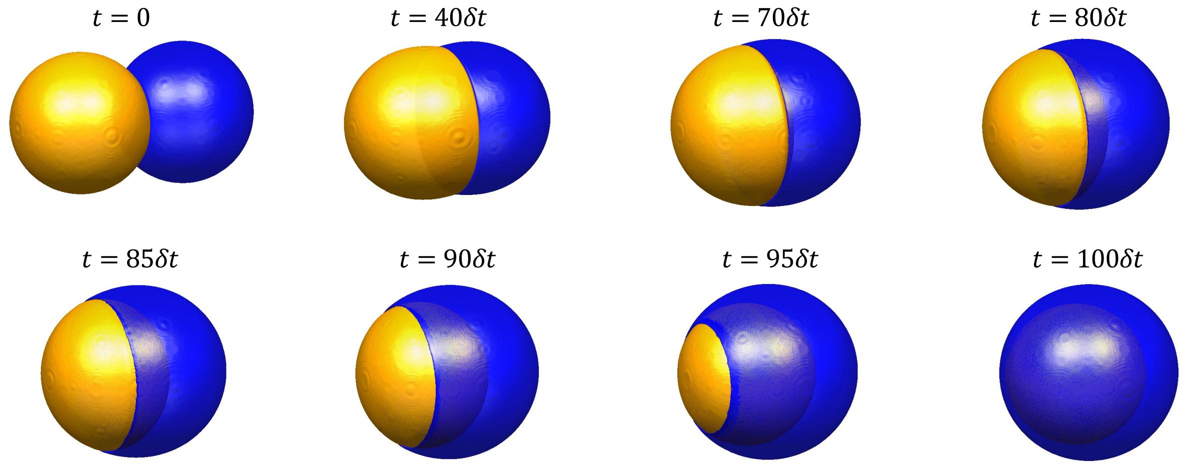

An initial cell doublet configuration ($t = 0$) in three dimensions and snapshots of its evolution using the level set-based approach, with time step $\delta t = 0.005$ and interfacial tensions $\sigma_{\mathrm{BO}}, \sigma_{\mathrm{BW}}, \sigma_{\mathrm{OW}}$ following the setup in Maitre et al.[26], simulates an encapsulation phenomenon in embryo morphogenesis.

Supplementary Note 9. Further Simulations of Auditory Epithelium – Formation of Stable Pattern through Fluctuations

This note provides more details on the simulation of developing auditory epithelium regarding its dependence on the choice of initial condition. It was found that perfect checkerboard or mosaic pattern was not reached for every initial configuration of HCs and SCs prescribed at E14 stage, even when simulation parameters described in the main text (Section 2.4) were used. Supplementary Figure 16a and Supplementary Movie 14 show numerical results obtained from a biased initial configuration where HCs and SCs cluster, contrary to the initial configuration in the main text (Figure 5c). The interfacial tensions are identical to those in the simulation shown in the main text (Figure 5e), i.e., $\sigma_{\mathrm{SS}} = \sigma_{\mathrm{SH}} = \sigma_{\mathrm{HH}} = \sigma_{\mathrm{SP}} = \sigma_{\mathrm{HP}} = 1.0$ at E14 stage, only σ_{SH} changes to 0.6 at E16 stage, and finally $\sigma_{\mathrm{SS}} = 0.3$, $\sigma_{\mathrm{SH}} = 0.6$, $\sigma_{\mathrm{HH}} = 1.0$, $\sigma_{\mathrm{SP}} = 0.65$ and $\sigma_{\mathrm{HP}} = 1.0$ at E18 stage. Under this setup, cells did not reach perfect checkerboard pattern at E16 and the final pattern at E18 was not the expected regular mosaic either.

Supplementary Figure 16: Simulation of developing auditory epithelium starting from a biased initial state and impact of fluctuations.

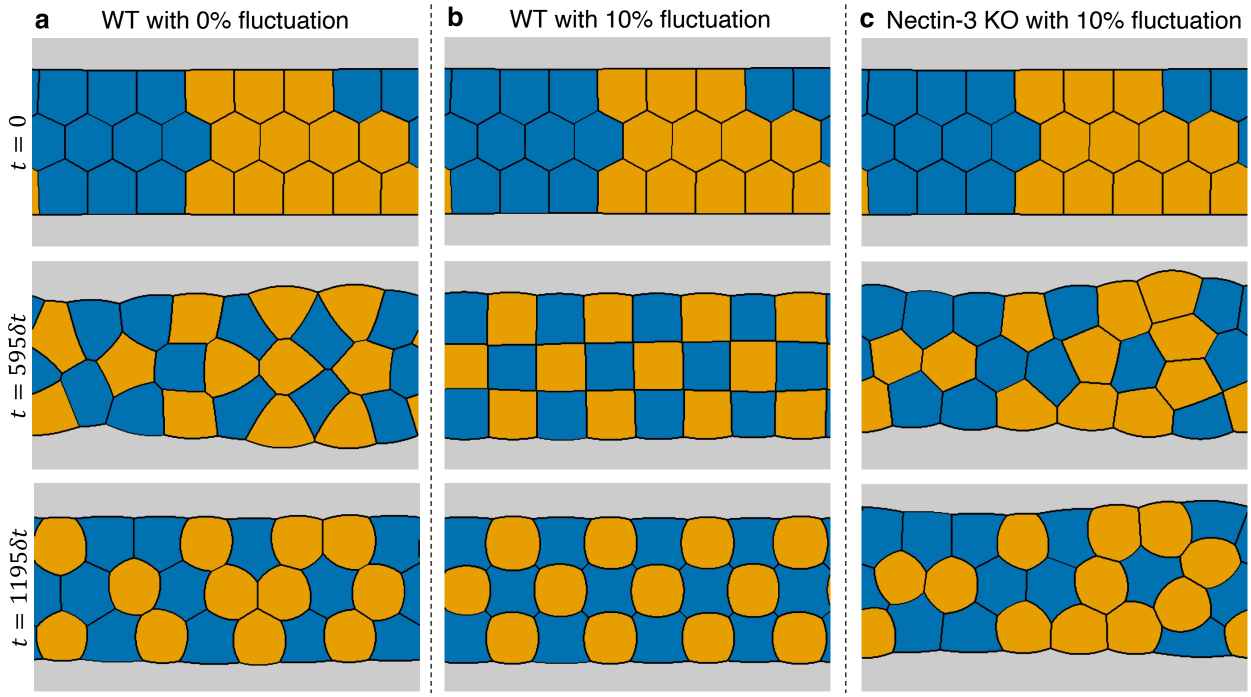

a, Level set-based simulation of cellular rearrangements of WT embryonic auditory epithelium (cf., Section 2.4 of the main text) starting from a biased initial configuration of 12 HCs (orange), 12 SCs (blue), and one pillar cell (gray) did not reach the expected mosaic pattern. **b**, On the other hand, weak fluctuations in surface tensions (within 10% of the original value) led to the stable attainment of regular patterns at E16 and E18 stages. **c**, The same fluctuations as in **b** did not improve the impaired final pattern obtained under the Nectin-3 KO setup.

In living tissues, various factors such as cellular fluctuations or mechanical forces[27], induce relocation of cells. Supplementary Figure 16b shows a simulation where a weak fluctuation within the range of 10% of the form

$$\tilde{\sigma}^{ij}_{\mathrm{AB}}(t) = \left(1 + 0.1\ \min\{1, e^{-16(t-1)}\} \sin \frac{\mathrm{mod}(7i + 9j, 16N)}{N} t\right) \sigma_{\mathrm{AB}}. \tag{S.3}$$

was imposed on the interfacial tensions. Here, $\tilde{\sigma}^{ij}_{\mathrm{AB}}$ is the modified interfacial tension between two cells with numbers i and j, symbols A and B are to be replaced by any of the symbols H, S or P, and $N = 25$ is the number of cells. In this case, perfect checkerboard pattern at E16 and the expected mosaic pattern

at E18 were obtained. This simulation indicates that, independently of the cell configuration before stage E14, the combination of the affinity between HCs and SCs at stage E16 and of the fluctuations lead to a regular checkerboard pattern and subsequently to the desired highly organized pattern at E18. Elucidation of the mechanism of pattern formation requires further investigation and our level set-based approach can be a reliable tool for pursuing this research. Supplementary Figure 16c and Supplementary Movie 14 depict results for the Nectin-3 KO simulation, i.e., $\sigma_{\mathrm{SH}} = 0.9$ at E16. In this case, checkerboard pattern is not achieved at E16, even if fluctuations are imparted to interfacial tensions, and one confirms that the resulting final pattern coincides with the experimentally observed one (see Figure 5f in the main text).

Supplementary References

[1] Armstrong, N. J., Painter, K. J., and Sherratt, J. A. A continuum approach to modelling cell-cell adhesion. *J. Theor. Biol.*, 243(1):98–113, 2006. doi:10.1016/j.jtbi.2006.05.030.

[2] Carrillo, J. A., Murakawa, H., Sato, M., et al. A population dynamics model of cell-cell adhesion incorporating population pressure and density saturation. *Journal of Theoretical Biology*, 474(1):14–24, 2019. doi:10.1016/j.jtbi.2019.04.023.

[3] Nagai, T. and Honda, H. A dynamic cell model for the formation of epithelial tissues. *Philosophical Magazine B*, 81(7):699–719, 07 2001. doi:10.1080/13642810108205772.

[4] Fletcher, A. G., Osborne, J. M., Maini, P. K., and Gavaghan, D. J. Implementing vertex dynamics models of cell populations in biology within a consistent computational framework. *Progress in Biophysics and Molecular Biology*, 113(2):299–326, 2013. doi:10.1016/j.pbiomolbio.2013.09.003.

[5] Ishimoto, Y. and Morishita, Y. Bubbly vertex dynamics: A dynamical and geometrical model for epithelial tissues with curved cell shapes. *Physial Review E*, 90:052711, 2014. doi:10.1103/PhysRevE.90.052711.

[6] Glazier, J. A. and Graner, F. Simulation of the differential adhesion driven rearrangement of biological cells. *Phys. Rev. E*, 47:2128–2154, 03 1993. doi:10.1103/PhysRevE.47.2128.

[7] Brodland, G. W. Computational modeling of cell sorting, tissue engulfment, and related phenomena: A review. *Applied Mechanics Reviews*, 57(1):47–76, 2004. doi:10.1115/1.1583758.

[8] Chen, H. H. and Brodland, G. W. Cell-level finite element studies of viscous cells in planar aggregates. *Journal of Biomechanical Engineering*, 122(4):394–401, 03 2000. doi:10.1115/1.1286563.

[9] Zhao, J., Cao, Y., DiPietro, L. A., and Liang, J. Dynamic cellular finite-element method for modelling large-scale cell migration and proliferation under the control of mechanical and biochemical cues: a study of re-epithelialization. *J. R. Soc. Interface*, 14:20160959, 2017. doi:10.1098/rsif.2016.0959.

[10] Brakke, K. A. Surface Evolver, 2013. http://facstaff.susqu.edu/b/brakke/evolver/evolver.html.

[11] Steinberg, M. S. Reconstruction of tissues by dissociated cells: Some morphogenetic tissue movements and the sorting out of embryonic cells may have a common explanation. *Science*, 141(3579):401–408, 1963. doi:10.1126/science.141.3579.401.

[12] Foty, R. A. and Steinberg, M. S. The differential adhesion hypothesis: a direct evaluation. *Developmental Biology*, 278(1):255–263, 2005. doi:10.1016/j.ydbio.2004.11.012.

[13] Brodland, G. W. The differential interfacial tension hypothesis (DITH): A comprehensive theory for the self-rearrangement of embryonic cells and tissues. *Journal of Biomechanical Engineering*, 124(2):188–197, 2002. doi:10.1115/1.1449491.

[14] Harris, A. K. Is cell sorting caused by differences in the work of intercellular adhesion? A critique of the Steinberg hypothesis. *Journal of Theoretical Biology*, 61(2):267–285, 1976. doi:10.1016/0022-5193(76)90019-9.

[15] Maître, J.-L., Niwayama, R., Turlier, H., et al. Pulsatile cell-autonomous contractility drives compaction in the mouse embryo. *Nature Cell Biology*, 17(7):849–855, 06 2015. doi:10.1038/ncb3185.

[16] Katsunuma, S., Honda, H., Shinoda, T., et al. Synergistic action of nectins and cadherins generates the mosaic cellular pattern of the olfactory epithelium. *The Journal of Cell Biology*, 212(5):561–575, 02 2016. doi:10.1083/jcb.201509020.

[17] Maître, J.-L. and Heisenberg, C.-P. The role of adhesion energy in controlling cell–cell contacts. *Current Opinion in Cell Biology*, 23(5):508–514, 2011. doi:10.1016/j.ceb.2011.07.004.

[18] Esedoḡlu, S. and Otto, F. Threshold dynamics for networks with arbitrary surface tensions. *Communications on Pure and Applied Mathematics*, 68(5):808–864, 2015. doi:10.1002/cpa.21527.

[19] Salvador, T. and Esedoḡlu, S. A simplified threshold dynamics algorithm for isotropic surface energies. *Journal of Scientific Computing*, 79(1):648–669, 2019. doi:10.1007/s10915-018-0866-8.

[20] Jacobs, M., Merkurjev, E., and Esedoḡlu, S. Auction dynamics: A volume constrained MBO scheme. *Journal of Computational Physics*, 354:288–310, 2018. doi:10.1016/j.jcp.2017.10.036.

[21] Laux, T. and Otto, F. Convergence of the thresholding scheme for multi-phase mean-curvature flow. *Calculus of Variations and Partial Differential Equations*, 55:129, 2016. doi:10.1007/s00526-016-1053-0.

[22] Nose, A., Nagafuchi, A., and Takeichi, M. Expressed recombinant cadherins mediate cell sorting in model systems. *Cell*, 54(7):993–1001, 1988. doi:10.1016/0092-8674(88)90114-6.

[23] Steinberg, M. S. and Takeichi, M. Experimental specification of cell sorting, tissue spreading, and specific spatial patterning by quantitative differences in cadherin expression. *Proceedings of the National Academy of Sciences of the United States of America*, 91(1):206–209, 1994. doi:10.1073/pnas.91.1.206.

[24] Togashi, H., Kominami, K., Waseda, M., et al. Nectins establish a checkerboard-like cellular pattern in the auditory epithelium. *Science*, 333(6046):1144–1147, 08 2011. doi:10.1126/science.1208467.

[25] Brodland, G. W. and Chen, H. H. The mechanics of heterotypic cell aggregates: Insights from computer simulations. *Journal of Biomechanical Engineering*, 122(4):402–407, 2000. doi:10.1115/1.1288205.

[26] Maître, J.-L., Turlier, H., Illukkumbura, R., et al. Asymmetric division of contractile domains couples cell positioning and fate specification. *Nature*, 536:344–348, 08 2016. 10.1038/nature18958.

[27] Cohen, R., Amir-Zilberstein, L., Hersch, M., et al. Mechanical forces drive ordered patterning of hair cells in the mammalian inner ear. *Nature Communications*, 11, 10 2020. doi:10.1038/s41467-020-18894-8.
